# Differential associations of allergic disease genetic variants with developmental profiles of eczema, wheeze and rhinitis

**DOI:** 10.1111/cea.13485

**Published:** 2019-10-15

**Authors:** Hannah Clark, Raquel Granell, John A. Curtin, Danielle Belgrave, Angela Simpson, Clare Murray, A. John Henderson, Adnan Custovic, Lavinia Paternoster

**Affiliations:** ^1^ MRC Integrative Epidemiology Unit (IEU) Population Health Sciences Bristol Medical School University of Bristol Bristol UK; ^2^ Division of Infection, Immunity and Respiratory Medicine School of Biological Sciences The University of Manchester Manchester Academic Health Science Centre, and Manchester University NHS Foundation Trust Manchester UK; ^3^ Section of Paediatrics Department of Medicine Imperial College London London UK

**Keywords:** asthma, atopic dermatitis, Avon Longitudinal Study of Parents and Children, eczema, genetics, rhinitis

## Abstract

**Background:**

Allergic diseases (eczema, wheeze and rhinitis) in children often present as heterogeneous phenotypes. Understanding genetic associations of specific patterns of symptoms might facilitate understanding of the underlying biological mechanisms.

**Objective:**

To examine associations between allergic disease‐related variants identified in a recent genome‐wide association study and latent classes of allergic diseases (LCADs) in two population‐based birth cohorts.

**Methods:**

Eight previously defined LCADs between birth and 11 years: “No disease,” “Atopic march,” “Persistent eczema and wheeze,” “Persistent eczema with later‐onset rhinitis,” “Persistent wheeze with later‐onset rhinitis,” “Transient wheeze,” “Eczema only” and “Rhinitis only” were used as the study outcome. Weighted multinomial logistic regression was used to estimate associations between 135 SNPs (and a polygenic risk score, PRS) and LCADs among 6345 individuals from The Avon Longitudinal Study of Parents and Children (ALSPAC). Heterogeneity across LCADs was assessed before and after Bonferroni correction. Results were replicated in Manchester Asthma and Allergy Study (MAAS) (n = 896) and pooled in a meta‐analysis.

**Results:**

We found strong evidence for differential genetic associations across the LCADs; pooled PRS heterogeneity *P*‐value = 3.3 × 10^−14^, excluding “no disease” class. The associations between the PRS and LCADs in MAAS were remarkably similar to ALSPAC. Two SNPs (a protein‐truncating variant in *FLG* and a SNP within an intron of *GSDMB*) had evidence for differential association (pooled *P*‐values ≤ 0.006). The *FLG* locus was differentially associated across LCADs that included eczema, with stronger associations for LCADs with comorbid wheeze and rhinitis. The *GSDMB* locus in contrast was equally associated across LCADs that included wheeze.

**Conclusions and clinical relevance:**

We have shown complex, but distinct patterns of genetic associations with LCADs, suggesting that heterogeneous mechanisms underlie individual disease trajectories. Establishing the combination of allergic diseases with which each genetic variant is associated may inform therapeutic development and/or predictive modelling.

## 1 INTRODUCTION

Although asthma, eczema (atopic dermatitis) and allergic rhinitis (AR) are diagnosed clinically as distinct conditions, their co‐occurrence is well acknowledged.^1^ Twin heritability estimates vary vastly, the range has been reported to be between 35% and 95% for asthma, 33% and 91% for AR and 71% and 84% for eczema,^2^ and estimates of the genetic correlation between them are also high: 0.55 between asthma and eczema, 0.47 between asthma and rhinitis and 0.62 between eczema and rhinitis.^3^ This supports shared genetic factors between all three conditions, but also genetic factors that are specific to each. Although we are starting to uncover both shared and specific genetic risk factors, understanding more precisely which loci predispose individuals to particular patterns of symptoms should translate into clinical benefits, such as personalized approaches to disease management and identification of novel therapeutic targets.^4^

Children often present with broad and heterogeneous phenotypes of allergic diseases.^5^, ^6^ For example, some cases have mild symptoms affecting a single organ/system, but others have more severe symptoms encompassing multiple organs (eg skin, upper and lower airways). The term “atopic march” has been proposed for a specific pattern of progression from childhood eczema to subsequent asthma and AR.^7^ We have previously used Bayesian machine learning methods to model the developmental profiles of eczema, wheeze and rhinitis during childhood in two population‐based birth cohorts: the Avon Longitudinal Study of Parent and Children (ALSPAC) and the Manchester Asthma and Allergy Study (MAAS).^8^ Eight latent classes were identified, each characterized by unique patterns of diseases over time: “No disease” (51.3% of the children), “Atopic march” (3.1%), “Persistent eczema and wheeze” (2.7%), “Persistent eczema with later‐onset rhinitis” (4.7%), “Persistent wheeze with later‐onset rhinitis” (5.7%), “Transient wheeze” (7.7%), “Eczema only” (15.3%) and “Rhinitis only” (9.6%).

A recent genome wide association study (GWAS)^9^ identified 136 independent risk variants (*P* < 3 × 10^−8^) for asthma, eczema and AR, including 73 novel variants, and went on to link the pathophysiology of allergic diseases to 132 neighbouring genes. Although this study was designed to detect risk variants shared across allergic diseases, six SNPs in five established risk loci (rs61816761, rs921650, rs115288876, rs12470864, rs6594499 and rs61839660) were identified as having some disease‐specific effects. In this study, we aimed to examine the associations between these 136 genetic variants associated with allergic diseases^9^ and the developmental profiles of eczema, wheeze, and rhinitis (“Latent Classes of Allergic Diseases”, LCADs) we previously described^8^ to better understand the genetic heterogeneity between the allergic disease class profiles.

## 2 METHODS

### 2.1 Study populations

Avon Longitudinal Study of Parents and Children is a population‐based birth cohort which recruited 14 541 pregnant women with expected delivery dates between 1st April 1991 and 31st December 1992. The study protocol was described previously.^10^, ^11^ Ethical approval was obtained from the ALSPAC Ethics and Law Committee and local research ethics committees. The study website contains details of all the data that are available through a fully searchable data dictionary: http://www.bris.ac.uk/alspac/researchers/data-access/data-dictionary/.

Manchester Asthma and Allergy Study is an unselected birth cohort established in 1995 in Manchester.^12^ It included 1184 children born between February 1996 and April 1998. Participants were recruited prenatally and followed prospectively.^13^ The study was approved by the Local Research Ethics Committee.

### 2.2 Latent classes of allergic diseases (LCADs)

Eight LCADs were previously identified using a latent disease profile model in a Bayesian machine learning modelling framework applied to longitudinal individual reports of eczema, wheezing and rhinitis collected at multiple time points between ages 1 and 11 years using joint ALSPAC and MAAS data^8^, ^14^: “No disease”, “Atopic march,” “Persistent eczema and wheeze,” “Persistent eczema with later‐onset rhinitis”, “Persistent wheeze with later‐onset rhinitis,” “Transient wheeze,” “Eczema only,” “Rhinitis only”; these are described in more detail in the Supplemental Material. Posterior probabilities of class membership (mean probabilities between 0.76 and 0.94 in joint ALSPAC and MAAS data) were used as the outcome in this study (Table S2 in Belgrave et al^8^).

### 2.3 Genotyping

In ALSPAC, we used the Illumina HumanHap550 quad chip and imputed against the Dec 2013 Version 1 Phase 3 release of 1000 Genomes reference haplotypes^15^ using IMPUTE2.^16^ Participants with genetic evidence of non‐European ancestry were excluded before imputation. Further details describing the QC and imputation procedure of the ALSPAC genetic data can be found here.^17^ In MAAS, DNA samples were genotyped using Illumina 610 quad and imputed using the 1000 Genomes Phase 3 integrated variant set reference.^18^

#### 2.3.1 Genetic risk score

Per‐allele SNP dosages were extracted in ALSPAC for the 136 SNPs identified in previous GWAS,^9^ and proxies were identified for SNPs with imputation quality < 0.80 (Table S1). All 136 relevant SNPs were available; SNP rs34290285 was excluded due to poor imputation quality and lack of adequate proxy. All 135 SNPs included in the ALSPAC score were available in MAAS; SNP rs10305290 was monomorphic and hence excluded from the MAAS score (see Supplemental Material). A weighted polygenic risk score (PRS) including 135 SNPs with INFO ≥0.77 was derived, weighted according to the overall effect sizes observed in Ref. 9. The standardized PRS represented a per 1‐standard deviation increase in the weighted risk score (details in Supplemental Material). Individual SNPs were also analysed separately, where each SNP was coded as the dosage of the risk allele. Following investigation of individual SNPs, a modified PRS, which excluded any SNPs with evidence for differential association, was generated to test for any residual effects among the remaining SNPs.

### 2.4 Association analysis

For the PRS and each SNP, a multinomial logistic regression was conducted, and relative risk ratios (RRR; also known as multinomial odd ratio) with corresponding 95% confidence intervals (Cis) for associations of the PRS or SNP dosages with the LCADs were reported. All regressions were weighted by posterior membership probabilities (the probabilities that a child belongs to each class) to account for uncertainty in class membership. “No disease” class was used as the baseline group.

Heterogeneity *P*‐values from chi‐square tests excluding (degrees of freedom = 6) and including (degrees of freedom = 7) the “No disease” class were generated (using post‐estimation *test* command) to test for both associations with any latent class and differential associations across LCADs. To further assess heterogeneity, pairwise tests among disease latent classes were performed when omnibus test for differential associations provided nominal evidence for overall heterogeneity.

In the single SNP analysis, a Bonferroni‐corrected *P*‐value threshold was calculated (*P* = .05/135 = 3.7 × 10^−4^) to control for type 1 error. For six SNPs reported as disease‐specific in GWAS (rs61816761, rs921650, rs115288876, rs12470864, rs6594499 and rs61839660),^9^ a less stringent p‐value threshold of 8.3 × 10^−3^ (0.05/6) was also used.

To assess the level of power in the analysis of ALSPAC data, power calculations were conducted for associations with individual phenotypes (details in the Supplemental Material and Table S2).

The associations of the nominal SNPs (*P*  < .05) identified in ALSPAC and the genetic risk scores (original and modified versions) with LCADs were then tested in MAAS. Pooled estimates and overall test for heterogeneity between sub‐groups (including and excluding “No disease” class) were derived. All statistical analyses were conducted with Stata 15.0.^19^

## 3 RESULTS

### 3.1 Characteristics of the study populations

A total of 6345 participants in ALSPAC and 896 in MAAS had both genetic and outcome data. Characteristics of the study populations are presented in Table 1.

**Table 1:** Characteristics of the study populations in ALSPAC and MAAS cohorts

Characteristics	Study sample^d^		Excluded sample (no data on outcome and/or genetics)		*P*‐value^c^
	N with characteristic (%)	Total N	N with characteristic (%)	Total N	
ALSPAC					
Gender (male)	3244 (51.1)	6345	4375 (51.6)	8479	0.575
Lower combined social class^a^	2617 (43.9)	5957	3017 (54.4)	5547	3.54E‐29
Lower maternal educational level^b^	3577 (57.5)	6220	4466 (71.8)	6218	1.31E‐62
Allergic diseases latent classes^e^					
No Disease	3175 (50.0)	6345	1150 (49.8)	2309	(Reference)
Atopic March	175 (2.8)		59 (2.5)		0.621
Persistent eczema and wheeze	181 (2.9)		72 (3.1)		0.508
Persistent eczema with late‐onset rhinitis	307 (4.8)		111 (4.8)		0.991
Persistent wheeze with late‐onset rhinitis	348 (5.5)		149 (6.5)		0.107
Transient wheeze	534 (8.4)		195 (8.4)		0.939
Eczema only	948 (15.0)		323 (14.0)		0.394
Rhinitis only	677 (10.7)		250 (10.8)		0.820
MAAS					
Gender (male)	482 (53.8)	896	186 (55.7)	287	0.305
Lower combined social class^a^	225 (36.9)	610	60 (43.2)	139	0.101
Lower maternal educational level^b^	359 (55.3)	649	45 (57.7)	78	0.392
Allergic diseases latent classes^e^					
No Disease	322 (35.9)	896	87 (38.9)	223	(reference)
Atopic March	55 (6.1)		11 (5.1)		0.471
Persistent eczema and wheeze	46 (5.1)		10 (4.6)		0.611
Persistent eczema with late‐onset rhinitis	73 (8.1)		18 (7.9)		0.710
Persistent wheeze with late‐onset rhinitis	81 (9.0)		18 (8.2)		0.557
Transient wheeze	65 (7.3)		15 (6.8)		0.644
Eczema only	141 (15.7)		34 (15.3)		0.634
Rhinitis only	114 (12.8)		29 (13.1)		0.836

a Mother and partner level of occupation (manual vs non‐manual) in ALSPAC, combined socio‐economic status (not working, routine, intermediate vs managerial) in MAAS.

b In ALSPAC, mother educated to the General Certificate of Education level (school‐leaving certificate) or lower; In MAAS, mother not educated to UK A or As level (data from follow‐up questionnaire at 13‐16 y).

c *P*‐values are from chi‐squared test for binary variables and logistic regression for latent variables (baseline group is “no disease” class), comparing “included” vs “excluded” individuals.

d Participants with SNP data and outcome data available.

e Proportions were weighted by posterior membership probabilities.

Similar proportions of males (51.1%, 53.8%), lower social class (43.9%, 36.9%) and lower maternal educational level (57.5%, 55.3%) were reported in ALSPAC and MAAS, respectively (for more details see Supplemental Material). Parents of participants excluded from the study because of missing data tended to be from the lower social class (*P* = 3.54 × 10^−29^) and lower maternal educational level (*P* = 1.31 × 10^−62^) in ALSPAC, as seen before.^20^ Marginal evidence for lower social class among the excluded individual was observed in MAAS (*P* = .10), but not for lower maternal educational level (*P* = .39). No differences were found between proportions of LCADs in the study populations *vs*. the excluded samples in either cohort (Table 1).

### 3.2 Discovery population (ALSPAC)

#### 3.2.1 Standardized genetic score (original)

**Figure 1:**
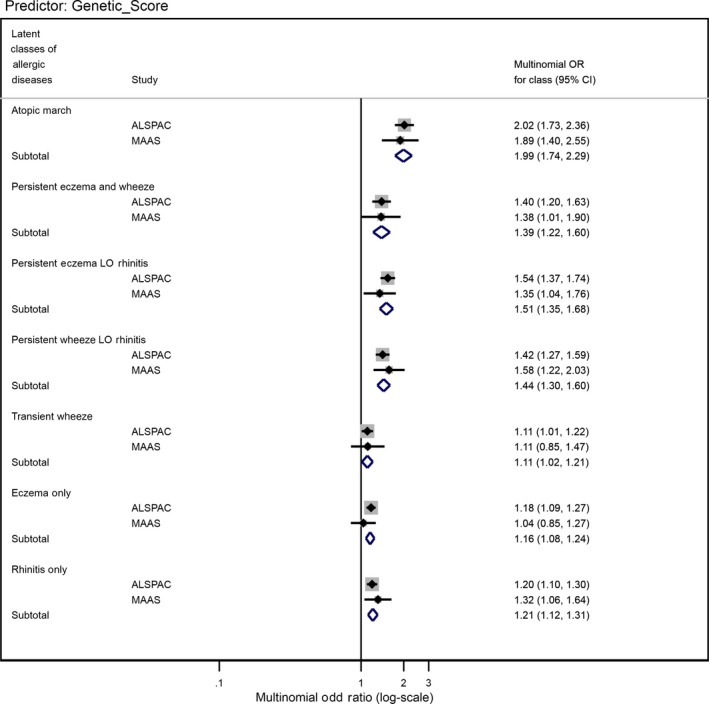
Forest plot of the associations between the standardized genetic score and allergic disease latent classes in ALSPAC and MAAS cohorts

There was strong evidence for an association between the standardized PRS and the LCADs (het. *P*‐value = 3.2 × 10^−30^ including “No disease” class) and strong evidence for differential association with the PRS across the latent classes (het *P*‐value = 3.3 × 10^−13^ excluding “No disease” class), with stronger associations observed for classes with more than one disease (Figure 1 and Table S3). Pairwise tests provide further evidence for differential associations between multiple‐disease and single‐disease classes: *P*‐values ≤ 9.68 × 10^−10^ for “atopic march” vs “transient wheeze,” “eczema only” and “rhinitis only” (Table S4).

#### 3.2.2 Individual SNP associations

**Figure 2:**
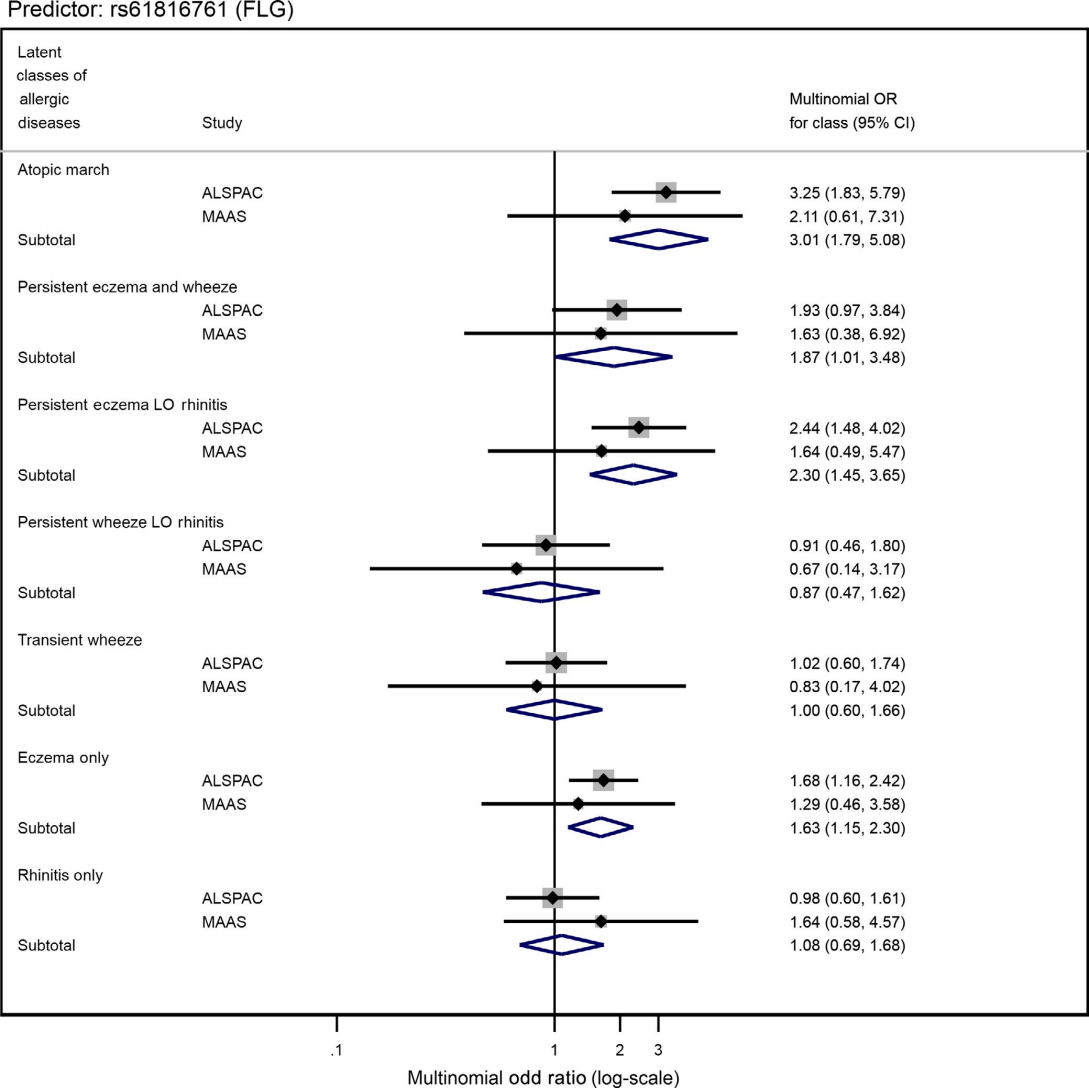
Forest plot of the associations between SNP rs61816761[A] near *Filaggrin* gene and allergic disease latent classes in ALSPAC and MAAS cohorts

**Figure 3:**
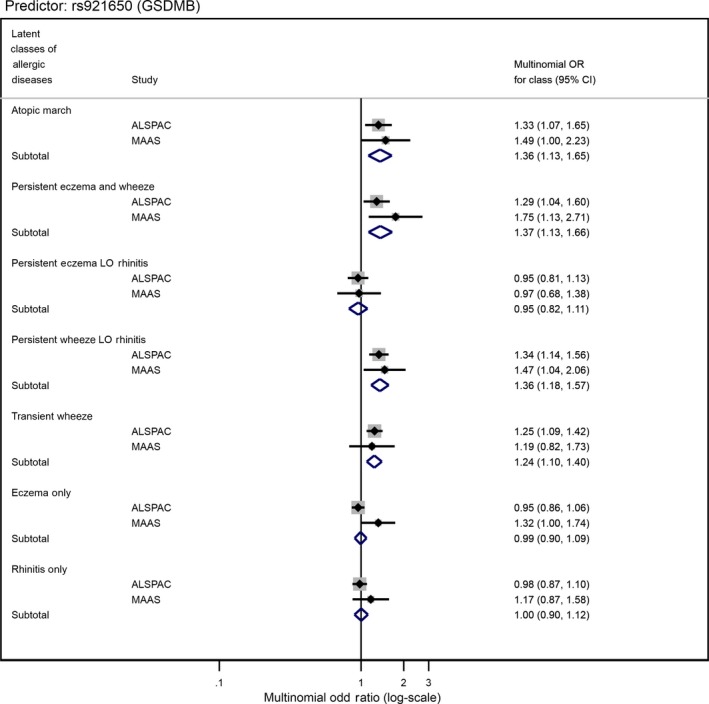
Forest plot of the associations between SNP rs921650[A] near *GSDMB* gene and allergic disease latent classes in ALSPAC and MAAS cohorts

Table S5 reports individual associations for all 135 individual SNPs with LCADs. Two SNPs, rs61816761 (a protein‐truncating variant in *FLG*) and rs921650 (within an intron of *GSDMB*), showed strong evidence for differential association between the LCADs (*P*‐values 0.002 and 1.42 × 10^−5,^ respectively, excluding “No disease” class) (Table S5 and Figures 2 and 3). Pairwise tests provide further evidence of these differential associations: *P*‐values ≤ 8.8 × 10^−3^ for “transient wheeze” vs “persistent eczema LO rhinitis”, “eczema only” and “rhinitis only” in the association between rs921650 (*GSDMB*) and the LCADs; *P*‐values ≤ 3.0 × 10^−3^ for “atopic march” vs. “persistent wheeze LO rhinitis,” “transient wheeze” and “rhinitis only” in the associations between rs61816761 (*FLG*) and the LCADs (Table S6).

**Figure 4:**
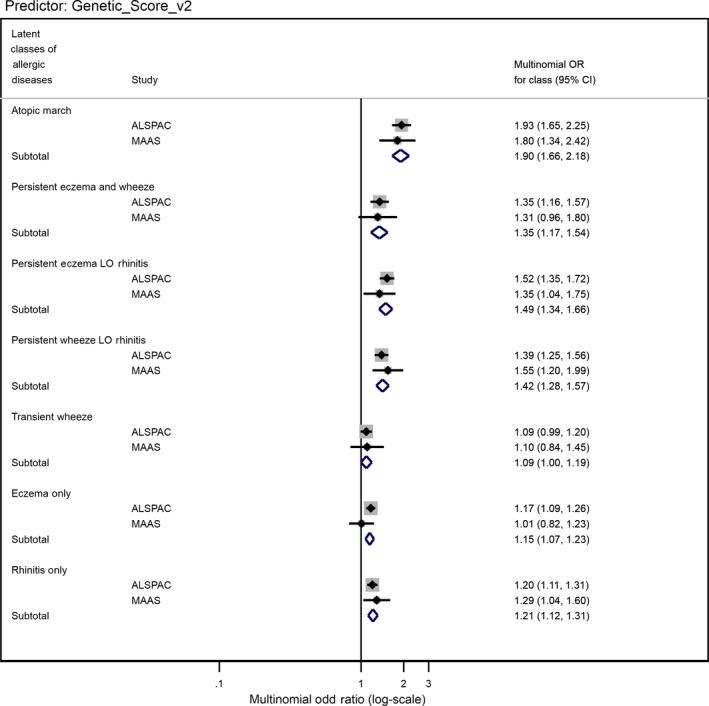
Forest plot of the associations between the modified standardized genetic score (excluding SNPs rs61816761 and rs921650) and allergic disease latent classes in ALSPAC and MAAS cohorts

A modified PRS which excluded these two SNPs still showed strong evidence for differential association (heterogeneity *P*‐value = 9.6 × 10^−12^, Table S3; Figure 4), suggesting that there are additional differential associations among the SNPs which did not meet our significance threshold.

Four additional SNPs (rs11652139, rs479844, rs6990534 and rs5743618) showed nominal evidence of differential association across the LCADs (heterogeneity *P*‐values < .05, Table S5). Pairwise tests provide further evidence of these differential associations. For example: *P*‐values ≤ 6.8 × 10^−3^ for “eczema only” vs “atopic march,” “persistent wheeze LO rhinitis” and “transient wheeze” in the association between rs11652139 and the LCADs; *P*‐values ≤ .007 for “atopic march” vs “transient wheeze” and “eczema only” in the association between rs6990534 and the LCADs (Table S6).

### 3.3 Replication in MAAS and Pooled Estimates

#### 3.3.1 Standardized genetic scores (original and modified)

The associations between the PRS and LCADs in MAAS were remarkably similar to ALSPAC (Table S7 and Figure 1). In the pooled analysis, there was strong evidence for differential association across LCADs (excluding the baseline class; *P*‐value = 3.27 × 10^−14^). To investigate the nature of these differences, we compared ORs and CIs across the seven classes. There was evidence for a large effect on “Atopic march” (pooled RRR = 1.99, 95% CI 1.74‐2.29), “Persistent eczema with late‐onset rhinitis” (1.51, 1.35‐1.68), “Persistent wheeze with late‐onset rhinitis” (1.44, 1.30‐1.60) and “Persistent eczema and wheeze” (1.39, 1.22‐1.60), per 1‐SD increase in the per‐risk‐allele standardized PRS compared to “No disease”. A modest increased risk was observed for LCADs affecting single organ/system: “Transient wheeze” (1.11, 1.02‐1.21), “Eczema only” (1.16, 1.08‐1.24) and “Rhinitis only” (1.21, 1.12‐1.31), per unit increase in the PRS compared to “No disease” class (Table 2; Figure 1).

**Table 2:** ALSPAC‐MAAS pooled estimates of the associations between standardized genetic scores and profile latent classes before and after excluding SNPs rs61816761 (*FLG*) and rs921650 (*GSDMB*)

Allergic diseases latent classes	ALSPAC‐MAAS pooled multinomial odds ratio (95% CI)	
	Stand. Score	Stand. Score excluding *FLG* and *GSDMB* SNPs
Atopic March	1.99 (1.74,2.29)	1.90 (1.66,2.18)
Persistent eczema and wheeze	1.39 (1.22,1.60)	1.35 (1.17,1.54)
Persistent eczema with late‐onset rhinitis	1.51 (1.35,1.68)	1.49 (1.34,1.66)
Persistent wheeze with late‐onset rhinitis	1.44 (1.30,1.60)	1.42 (1.28,1.57)
Transient wheeze	1.11 (1.02,1.21)	1.09 (1.00,1.19)
Eczema only	1.16 (1.08,1.24)	1.15 (1.07,1.23)
Rhinitis only	1.21 (1.12,1.31)	1.21 (1.12,1.31)
P‐value for differential association^a^	3.27E‐14	1.07E‐12
P‐value for any association^b^	1.22E‐13	3.83E‐12

a Overall test for heterogeneity between classes excluding the baseline class of “no disease”.

b Overall test for heterogeneity between classes including the baseline class of “no disease” (Pooled multinomial Odds Ratio = 1).

Omitting SNPs rs61816761 (a protein‐truncating variant in *FLG*) and rs921650 (within an intron of *GSDMB*) from original standardized PRS slightly attenuated the associations across LCADs (pooled het. *P*‐value = 3.83 × 10^−12^ including “No disease,” 1.07 × 10^−12^ excluding “No disease” class) (Table 2; Figure 4).

#### 3.3.2 Individual SNP associations

We tested six nominally associated individual SNPs from ALSPAC (*P* < .05) in MAAS (Table S8). Pooled results are shown in Table 3. We found moderate evidence for differential associations across LCADs (excluding the baseline class) for SNPs rs61816761, rs921650 and rs11652139 (pooled heterogeneity *P*‐values ≤ .006). To investigate the nature of these differences, we compared ORs and CIs across the 7 LCADs. There was evidence for a large effect on “Atopic march” (pooled RRR = 3.01, 95%CI 1.79‐5.08), “Persistent eczema and wheeze” (1.87, 1.01‐3.48), “Persistent eczema with late‐onset rhinitis” (2.30, 1.45‐3.65) and “Eczema only” (1.63, 1.15‐2.30), per increased A‐allele of rs61816761 (a protein‐truncating variant in *FLG*) (Table 3; Figure 2). Little or no evidence of association was seen for other LCADs. We found evidence for a large effect on “Atopic march” (pooled RRR = 1.36, 95%CI 1.13‐1.65), “Persistent eczema and wheeze” (1.37, 1.13‐1.66), “Persistent wheeze with late‐onset rhinitis” (1.36, 1.18‐1.57) and “Transient wheeze” (1.24, 1.10‐1.40), per increased A‐allele of rs921650 (within an intron of *GSDMB*), compared to “No disease” (Table 3; Figure 3). Little or no evidence of association was seen for other LCADs.

**Table 3:** ALSPAC‐MAAS pooled estimates of the associations between the top six nominal SNPs (heterogeneity p‐value excluding the baseline “no disease” class < 0.05) and allergic diseases latent classes

SNP[RA]	ALSPAC‐MAAS pooled multinomial odds ratio (95% CI)					
	rs61816761[A]	rs921650[A]	rs11652139[A]	rs479844[G]	rs6990534[A]	rs5743618[C]
Nearby Gene	*FLG*	*GSDMB*				
Allergic diseases latent classes						
Atopic March	3.01 (1.79,5.08)	1.36 (1.13,1.65)	1.47 (1.20,1.80)	1.12 (0.92,1.36)	1.33 (1.09,1.62)	1.31 (1.04,1.67)
Persistent eczema and wheeze	1.87 (1.01,3.48)	1.37 (1.13,1.66)	1.22 (1.00,1.49)	1.19 (0.98,1.44)	0.92 (0.74,1.14)	0.98 (0.78,1.22)
Persistent eczema with late‐onset rhinitis	2.30 (1.45,3.65)	0.95 (0.82,1.11)	1.06 (0.91,1.24)	1.21 (1.04,1.41)	1.08 (0.91,1.27)	1.06 (0.89, 1.26)
Persistent wheeze with late‐onset rhinitis	0.87 (0.47,1.62)	1.36 (1.18,1.57)	1.22 (1.05,1.42)	0.95 (0.82,1.10)	1.03(0.88,1.21)	1.13 (0.96,1.35)
Transient wheeze	1.00 (0.60,1.66)	1.24 (1.10,1.40)	1.15 (1.02,1.31)	1.02 (0.90,1.16)	0.93 (0.81,1.07)	0.97 (0.84, 1.13)
Eczema only	1.63 (1.15,2.30)	0.99 (0.90,1.09)	0.98 (0.88,1.08)	1.14 (1.03,1.26)	0.96 (0.86,1.07)	0.99 (0.89, 1.11)
Rhinitis only	1.08 (0.69,1.68)	1.00 (0.90,1.12)	1.04 (0.93,1.17)	0.95 (0.85,1.06)	1.10 (0.98,1.24)	1.19 (1.04, 1.36)
*P*‐value for differential association^a^	0.006	1.71E‐05	0.006	0.044	0.055	0.138
*P*‐value for any association^b^	0.011	4.26E‐05	0.011	0.074	0.091	0.207

Abbreviation: RA, risk allele.

a Overall test for heterogeneity between classes excluding the baseline class of “no disease”.

b Overall test for heterogeneity between classes including the baseline class of “no disease” (Pooled multinomial Odds Ratio = 1).

The pattern of associations for SNP rs11652139 was very similar to SNP rs921650 (within an intron of *GSDMB*) (Table 3; Figure S1). For SNP rs479844, we found evidence for a moderate effect on “Persistent eczema with late‐onset rhinitis” (pooled RRR 1.21, 95% CI 1.04‐1.41) and “Eczema only” (1.14, 1.03‐1.26) per increased G‐allele, when compared to “No disease” class (Figure S2). We observed evidence for a moderate effect on “Atopic march” per increased A‐allele of SNP rs6990534 (1.33, 1.09‐1.62) (Figure S3) and per increased C‐allele of SNP rs5743618 (1.31, 1.04‐1.67); SNP rs5743618 showed evidence for a moderate effect on “Rhinitis only” (1.19, 1.04‐1.36) (Figure S4).

## 4 DISCUSSION

We examined the associations between 135 independent risk variants for allergic diseases identified in a recent GWAS^9^ and LCADs which we previously described in two independent birth cohorts.^8^ SNP rs61816761 (a protein‐truncating variant in *FLG* gene) and SNP rs921650 (within an intron of *GSDMB*) which were previously identified as having disease‐specific effects^9^ were differentially associated with distinct LCADs. The *FLG* locus was associated with all LCADs that included eczema, with stronger associations seen for those classes with comorbid wheeze and/or rhinitis. In contrast, the strength of the association for *GSDMB* locus was equal for all LCADs which included wheeze, with no additional risk for comorbid eczema and/or rhinitis. An allergic disease polygenic risk score (including all 135 SNPs and after excluding SNPs rs61816761 and rs921650) showed strong evidence of heterogeneity across the LCADs, suggesting there are further individual SNPs with differential association.

### 4.1 rs61816761 (*FLG)*

rs61816761 (also known as R501X) has long been linked with eczema.^21^ In Ferreira's GWAS,^9^ the risk allele [A] for SNP rs61816761, which is located on chromosome 1 in the *FLG* gene, was 1.32‐fold more common in individuals experiencing eczema‐only symptoms compared with individuals experiencing only AR. Similarly, a 1.26‐fold difference was observed when comparing individuals experiencing only eczema with individuals with only asthma. This indicated that this SNP predisposes to eczema more strongly than to either of the other two conditions. In the present study, we found evidence for a large effect on each of the four latent classes including eczema, but no association with classes which did not include eczema. We observed the strongest association with “Atopic march” (pooled RRR 3.01). This is in line with a recent meta‐analysis which found that the two *FLG* mutations combined (R501X and *FLG* 2282del4) were associated with Atopic march.^22^

It is tempting to speculate that genotyping patients with eczema for *FLG* mutations could help to identify individuals who may benefit from interventions targeted at prevention of progression to the atopic march.^23^ However, the difference in odds between the “Atopic march” versus “Eczema only” classes is of insufficient magnitude to be of a clinical predictive value. It is also important to note that whilst *FLG* mutations (including R501X) play a role in predisposing individuals of Caucasian ancestry to eczema, such mutations have not been seen in other ethnic groups.^24^

### 4.2 rs921650 (*GSDMB)*

In Ferreira's GWAS,^9^ the risk allele [A] for SNP rs921650 on chromosome 17q21.1 was 1.08‐fold more common in asthma‐only compared with eczema‐only cases. It was also 1.04‐fold more common in asthma‐only compared with AR‐only cases, thus representing a stronger risk factor for asthma compared with both eczema and AR. In the present study, we found evidence for a large effect on all four wheeze‐related classes per increased A‐allele of rs921650. However, unlike for associations with *FLG* locus, where we observed increasing odds for classes with comorbid wheeze and rhinitis (ORs from 1.87 to 3.01) when compared to “Eczema only” class (OR 1.63), for associations with *GSDMB* locus, we did not observe increasing odds for classes with comorbid eczema and rhinitis (ORs from 1.36 to 1.37), when compared to “Transient wheeze” class (OR 1.24). These results suggest that there is no additional risk of comorbid conditions compared to wheeze alone in association with *GSDMB*.

rs921650 is located within an intron of *GSDMB* (gasdermin B) gene (also known as *GSDML*),^25^, ^26^ likely affecting the expression of genes in the 17q21 locus. SNPs in the 17q21 locus have been associated with childhood asthma, and there is evidence that common SNPs in this region are associated with persistent wheeze.^27^ Results of our analysis are consistent with this evidence, with the increased risk of three classes characterized by wheeze persistence (“Persistent wheeze with late‐onset rhinitis,” “Persistent eczema and wheeze” and “Atopic march”) per increased A‐allele of rs921650. Our study has also shown evidence for an association of rs921650 with transient wheeze when defined as high probability of wheeze within the first 5 years, with remission by age 8 years, and a very low probability of eczema and rhinitis throughout childhood. Given that early‐life wheeze in the absence of other allergic symptoms (such as eczema) is mostly virus‐induced, our results are in agreement with observations from recent studies which provided evidence that polymorphisms on chromosome 17q21 regulate ICAM1 expression and thus may affect the frequency and severity of rhinovirus infection and early childhood wheezing illness,^28^ that impaired anti‐virus immunity is associated with early‐life wheezing,^29^ and that early‐life antibiotic use (a proxy for impaired innate anti‐virus immunity) is associated with 17q21 polymorphisms.^30^

### 4.3 Interpretation of the Polygenic Risk Score (PRS)

We report strong evidence of heterogeneity of associations across the LCADs (pooled het. *P*‐value = 3.3 × 10^−14^ excluding “No disease” class). The strongest association was seen for “Atopic march” (pooled RRR 1.99, 1.74‐2.29). Our results suggest that although the presence of multiple risk alleles increases the risk of all latent classes, this is strongest for the “Atopic march,” and weakest for the classes characterized by the presence of single symptoms. This may indicate that the greater the standardized per‐allele weighted PRS, the greater the likelihood of developing multiple comorbidities. After excluding SNPs rs61816761 and rs921650, this effect was still observed (pooled het. *P*‐value = 1.1 × 10^−12^). As none of these 133 individual SNPs showed good evidence for differential association, we can assume that there are some unidentified differential single SNP associations, which we did not have power to detect. Therefore, studies of larger sample sizes should be designed to investigate this further.

### 4.4 Strengths and Limitations

The prevalence of rhinitis‐related classes was higher in MAAS compared to ALSPAC.^8^ However, in the original study, similar latent profiles were identified in MAAS and ALSPAC, suggesting consistent patterns across the two populations.^8^ Furthermore, we have used latent classes derived from joint modelling, which accounted for these differences and increased the resolution of the identified latent classes.^8^

The restricted sample (after removal of individuals who had missing genetic and/or outcome data) resulted in a loss of participants from lower social class and maternal educational level. Selection bias can be introduced if the individuals from a lower social class had missing data because of factors related to the outcome. Issues such as this can also impact on the generalizability of the results. However, despite the loss of individuals from lower social class, there was still a good representation in the included study samples (43.9% in ALSPAC and 36.9% in MAAS).

Avon Longitudinal Study of Parents and Children was part of the meta‐analysis by Ferreira et al that was used to identify the 136 SNPs; this could have led to some overfitting in our ALSPAC analysis. However, our sample only makes up 0.6% of the sample used by Ferreira et al and our main analysis focuses on the comparison between case (disease) groups, rather than the comparison of combined cases versus controls (from which the beta values were taken) so any overfitting is likely to be negligible.

It is likely that combining the SNPs in a composite score increased the power sufficiently to detect an overall effect, even when individual SNPs showed little evidence of association. However, grouping together variants which may individually have different effects might act to attenuate or obscure individual effects.

Whilst understanding more about the role that the 135 genetic variants identified to date by Ferreira et al play in allergic disease profiles is beneficial, a limitation of the Ferreira approach is that it is better powered to detect homogenous as opposed to heterogeneous effects and so there may be additional SNPs with interesting heterogeneous effects that have yet to be identified. However, our results are a good proof of principle of the latent disease profile method as a good way to characterize heterogenous effects across the allergic disease classes. As carefully phenotyped GWAS sample sizes increase, it may be possible to undertake such latent disease profile analyses in a GWAS context with better power to detect novel loci with heterogenous effects.

### 4.5 Conclusions

We found strong evidence for differential genetic associations across different developmental profiles of eczema, wheeze and rhinitis, which were remarkably consistent across two cohorts. Two polymorphisms (a protein‐truncating variant in *FLG* and a SNP within an intron of *GSDMB*) showed evidence for distinct patterns of association. The *FLG* locus was associated with all profiles that included eczema, but with stronger associations for those with comorbid wheeze and/or rhinitis. The *GSDMB* locus in contrast was associated with all profiles which included wheeze (including wheezing up to age 5 years with remission by age 8 years), but with no additional risk of comorbid conditions. This emphasizes the likely complex and heterogeneous mechanisms underlying within‐individual disease trajectories and demonstrates the need for future studies to take account of the complex nature of these associations. Our analysis using a PRS also demonstrates that there is likely to be additional heterogeneity among other SNPs, that this study did not have power to detect. This approach to disentangling the complex nature of multi‐trait aetiology might be a promising one that should be used in future larger studies.

## Data Availability

The data that support the findings of this study are available from the corresponding author upon reasonable request.
