# Supplementary material for: Differential associations of allergic disease genetic variants with developmental profiles of eczema, wheeze and rhinitis
Source: Clin Exp Allergy. 2019 Oct 15;49(11):1475–86. doi: 10.1111/cea.13485 (PMC6899469; doi:10.1111/cea.13485)
Supplement: Supplementary file 1 [file CEA-49-1475-s001.docx]

**Differential associations of allergic disease genetic variants with developmental profiles of eczema, wheeze and rhinitis**

**Supplemental Material**

# Methods

## ALSPAC

Pregnant women resident in Avon, UK with expected dates of delivery 1st April 1991 to 31st December 1992 were invited to take part in the study. The initial number of pregnancies enrolled is 14,541 (for these at least one questionnaire has been returned or a “Children in Focus” clinic had been attended by 19/07/99). Of these initial pregnancies, there was a total of 14,676 foetuses, resulting in 14,062 live births and 13,988 children who were alive at 1 year of age.

When the oldest children were approximately 7 years of age, an attempt was made to bolster the initial sample with eligible cases who had failed to join the study originally. As a result, when considering variables collected from the age of seven onwards (and potentially abstracted from obstetric notes) there are data available for more than the 14,541 pregnancies mentioned above.

The number of new pregnancies not in the initial sample (known as Phase I enrolment) that are currently represented on the built files and reflecting enrolment status at the age of 24 is 904 (452, 254 and 198 recruited during Phases II, III and IV respectively), resulting in an additional 811 children being enrolled. The phases of enrolment are described in more detail in the cohort profile paper (see footnote 4 below). Please note that phase 4 enrolment (age 18-24) is not currently included in the cohort profile.

The total sample size for analyses using any data collected after the age of seven is therefore 15,247 pregnancies, resulting in 15,458 foetuses. Of this total sample of 15,656 foetuses, 14,973 were live births and 14,899 were alive at 1 year of age.

A 10% sample of the ALSPAC cohort, known as the Children in Focus (CiF) group, attended clinics at the University of Bristol at various time intervals between 4 to 61 months of age. The CiF group were chosen at random from the last 6 months of ALSPAC births (1432 families attended at least one clinic). Excluded were those mothers who had moved out of the area or were lost to follow-up, and those partaking in another study of infant development in Avon.

## Maternal educational attainment in the two cohorts

In ALSPAC, we obtained details of maternal educational attainment (lower level defined as educated to school leaving certificate at 16 years or lower), and joint parental social class (manual vs non-manual derived from occupation categorisation using the OPCS job codes) from questionnaires sent to the mother during pregnancy.

In MAAS, details on gender and socio-economic status (lower social class defined as not working, routine or intermediate; higher social class defined as managerial) were obtained from questionnaire data at recruitment. Maternal educational level was obtained from followed up questionnaires at age 13-16 years. Lower maternal education level was defined as not educated to UK A or As level.

## Imputation quality and frequency checks

The imputation quality of each SNP was assessed, and four SNPs (rs12123821, rs34290285, rs16903574, rs112401631) with imputation quality of <0.80 were investigated for potential SNP proxies with higher imputation quality. Proxies were identified using the LDproxy tool (LDlink) ([1](#_ENREF_1)) and selected based on highest INFO score and R² > 0.40 and D’ values >0.60 (for LD). The LDproxy tool was then used to identify the risk alleles for the proxies, so that the odds ratios (ORs) used for the proxy SNPs were the same as the top SNP (extracted from the GWAS paper ([2](#_ENREF_2))). Details on suitable proxies were found for three of the four SNPs and substituted into the dataset. No suitable proxy for rs34290285 was identified in ALSPAC, so this SNP was excluded from the dataset, leaving 135 SNPs in the ALSPAC analyses. All 135 SNPs were available in MAAS, however, SNP rs10305290 was monomorphic and hence excluded from the MAAS score.

In the dataset, the alleles for each SNP were coded as B1 and B2. The per-B2-allele dosages were extracted and checks were made to ensure that the SNP dosages were coded as expected before any further analysis took place, by comparing observed risk allele frequencies to those reported for 1000 Genome phase 3 in the Database of Single Nucleotide Polymorphisms (dbSNP) ([3](#_ENREF_3)). The formula used to calculate the allele frequencies in ALSPAC was:

$$\frac{\sumƊі}{ɳ\times2}$$

Where Ɗі was the per-B2-allele dosage for each SNP and ɳ was the number of individuals (8802). The comparison of ALSPAC and dbSNP frequencies showed that SNP dosages had been coded correctly. Per-allele SNP dosages were then flipped if necessary so that the dosages were coded as per risk-allele.

## Polygenic risk score (PRS)

A weighted polygenic risk score (PRS) was calculated for 8,802 individuals from ALSPAC with complete data on all 135 SNPs.

The following formula was used to calculate the PRS for each individual:

$$\frac{\sum(\betaі\timesƊі)}{ɳ}$$

Where βi is the beta value for each SNP (i.e. its effect size as reported by Ferreira et al. ([2](#_ENREF_2)) , converted by beta=log(OR)). Ɗi is the per risk-allele dosage of each SNP and ɳ is the total number of SNPs (135). The units of the derived risk score are per risk-allele weighted by effect size. A histogram was created to visualise the distribution of the score. A standardised version of the PRS was then calculated using the following formula:

$$\frac{\left( \frac{\sum(\betaі\timesƊі)}{ɳ} \right)-\bar{x}}{\sigma}$$

The standardised PRS represents a per 1-standard deviation increase in the per risk-allele weighted score. A histogram was also created for the standardised PRS. Information on the 135 SNPs used to generate the risk score (including their weight/beta values) can be found in **Table S1**.

## Allergic Diseases Latent Classes

The eight latent classes of allergic diseases were defined as described previously ([4](#_ENREF_4)):

1. No disease: children in this class had a low probability of eczema, wheeze and rhinitis
2. Atopic March: high probability of eczema from infancy to age 11 years with increased probability of wheeze over time. For rhinitis, the probability increased from zero at age 1 year to almost 100% by 8 years. Eczema developed first, followed by wheeze, and then rhinitis;
3. Persistent eczema and wheeze: similar probability of wheeze and eczema throughout childhood, likely as co-morbidities, with a low probability of rhinitis throughout childhood.
4. Persistent eczema with later-onset rhinitis (‘Persistent eczema later-onset rhinitis’): increased eczema prevalence from ~70% in early life to 95% at age 5 years, with little resolution at 11 years. The probability of rhinitis increased to almost 100% by age 8 years with low probability of wheeze throughout childhood.
5. Persistent wheeze with later-onset rhinitis (‘Persistent wheeze later-onset rhinitis’): high probability of wheeze throughout childhood, with increasing probability of rhinitis to almost 100% by age 11 years. Probability of eczema was low, declining steadily at age 11 years.
6. Transient wheeze: high probability of wheeze within the first 5 years, with remission by age 8 years, and a very low probability of eczema and rhinitis throughout childhood.
7. Eczema only: high probability of eczema throughout life, peaking at ~80% at age 5 years, then declining steadily to a 50% probability at age 11 years.
8. Rhinitis only: increasing probability of rhinitis from age 5 to 11 years, but no wheeze or eczema.

While in ALSPAC the ‘no disease’ class had a proportion of 50.0%, in MAAS this was only 36.5%. In both cohorts the largest disease latent classes were ‘eczema only’ (15.3% in ALSPAC vs. 15.6% in MAAS) and ‘rhinitis only’ (9.5% in ALSPAC vs. 12.8% in MAAS). ‘Atopic march’ (2.7% vs. 5.9%), ‘persistent eczema and wheeze’ (2.4% vs. 5.0%), ‘persistent eczema later-onset rhinitis’ (4.4% vs. 8.1%) and ‘persistent wheeze later-onset rhinitis’ (5.2% vs. 8.9%) were less prevalent in ALSPAC compared to MAAS. The proportion of ‘transient wheeze’ class was similar in both cohorts (7.7% vs. 7.2%).

## Power calculations in ALSPAC

To assess the level of power in the individual SNP analysis (the probability of detecting an effect given that the effect is real), power calculations were conducted for associations with individual phenotypes. Power calculations were performed using the GAS online power calculator ([5](#_ENREF_5))^.^  The calculations assumed an additive genetic model, with the following input parameters for each disease class: number of cases and controls, the significance level, prevalence, risk allele frequency and relative risk. The input ‘prevalence’ was taken from the previous publication ([4](#_ENREF_4)) on the latent disease classes, and the significance level was that of the Bonferroni corrected threshold. Calculations were made for various values of risk allele frequency (0.05, 0.15, 0.25, 0.45) and relative risk (1.1, 1.3, 1.5).

Results for the power calculations are displayed in **Table S2**. Generally, the power to detect associations for single SNPs in ALSPAC was low, especially for ‘Atopic march’ (n=173, power≤0.35), ’Persistent eczema and wheeze’ (n=154, power≤0.28) and ‘Persistent eczema with late-onset rhinitis’ (n=279, power≤0.70), where the sample size of each class was lower. As the effect size (RRR) increased and the risk allele frequency (RAF) increased, the power to detect associations also increased. Statistical power was >0.80 for detecting associations in ‘Persistent wheeze with late-onset rhinitis’ class (n=328) for minimum RRR 1.50 and minimum RAF 0.45. For ‘Transient wheeze’ (n=491) and ‘Rhinitis only’ (n=602) classes, power to detect associations above a level of 0.80 was reached at a minimum RRR 1.50 and minimum RAF 0.15. For ‘Eczema only’ class (n=971), the largest of the disease latent classes in terms of number of cases, power to detect associations above a level of 0.80 was reached at a minimum RRR 1.30 and minimum RAF 0.25.

# References

1. Machiela MJ, Chanock SJ. LDlink: a web-based application for exploring population-specific haplotype structure and linking correlated alleles of possible functional variants. Bioinformatics. 2015;31(21):3555-7.

2. Ferreira MA, Vonk JM, Baurecht H, Marenholz I, Tian C, Hoffman JD, et al. Shared genetic origin of asthma, hay fever and eczema elucidates allergic disease biology. Nat Genet. 2017;49(12):1752-7.

3. Sherry ST, Ward MH, Kholodov M, Baker J, Phan L, Smigielski EM, et al. dbSNP: the NCBI database of genetic variation. Nucleic Acids Res. 2001;29(1):308-11.

4. Belgrave DC, Granell R, Simpson A, Guiver J, Bishop C, Buchan I, et al. Developmental profiles of eczema, wheeze, and rhinitis: two population-based birth cohort studies. PLoS Med. 2014;11(10):e1001748.

5. Johnson JL CC, Li KW, Caron S, Abecasis G. . Genetic Association Study (GAS) Power Calculator 2017 [Available from: <http://csg.sph.umich.edu/abecasis/cats/gas_power_calculator/index.html>.

# Tables

## ALSPAC Results

### **Table S1.** Information on the 135 SNPs used to generate the standardised genetic score in ALSPAC cohort.

| **Chr** | **Position b37** | **SNP** | **Gene context** | **Risk allele** | **Other allele** | **Risk allele frequency** | **Info*** | **Weight†** | **Proxy for** | **Merged into‡** |
| --- | --- | --- | --- | --- | --- | --- | --- | --- | --- | --- |
| 1 | 2510755 | rs10910095 | *TNFRSF14-[]-FAM213B* | G | A | 0.865 | 0.983 | 0.041 |  |  |
| 1 | 8482078 | rs301806 | *[RERE]* | T | C | 0.581 | 0.997 | 0.047 |  |  |
| 1 | 25251923 | rs760805 | *[RUNX3]* | T | A | 0.57 | 0.986 | 0.037 |  |  |
| 1 | 35681738 | rs76167968 | *SFPQ-[]-ZMYM4* | T | C | 0.919 | 0.994 | 0.054 |  |  |
| 1 | 150265704 | rs7512552 | *C1orf54-[]-MRPS21* | C | T | 0.513 | 0.998 | 0.031 |  |  |
| 1 | 151796742 | rs11204896 | *[RORC]* | C | G | 0.899 | 0.974 | 0.061 |  |  |
| 1 | 152000117 | rs115288876 | *RPTN-[]-HRNR* | A | G | 0.053 | 0.765 | 0.105 | rs12123821 |  |
| 1 | 152285861 | rs61816761 | *[FLG]* | A | G | 0.021 | 0.803 | 0.202 |  |  |
| 1 | 154426970 | rs2228145 | *[IL6R]* | C | A | 0.416 | 0.999 | 0.037 |  |  |
| 1 | 161185058 | rs2070901 | *NDUFS2-[]-FCER1G* | T | G | 0.263 | 0.996 | 0.038 |  |  |
| 1 | 167431352 | rs2988277 | *[CD247]* | C | T | 0.607 | 0.991 | 0.039 |  |  |
| 1 | 172700868 | rs1102705 | *FASLG-[]--TNFSF18* | G | A | 0.101 | 0.954 | 0.056 |  |  |
| 1 | 173146921 | rs4090390 | *TNFSF18--[]-TNFSF4* | A | C | 0.239 | 0.952 | 0.047 |  |  |
| 1 | 226914734 | rs697852 | *[ITPKB]* | A | G | 0.819 | 0.974 | 0.040 |  |  |
| 2 | 8442248 | rs10174949 | *[LINC00299]* | G | A | 0.707 | 1 | 0.064 |  |  |
| 2 | 64836267 | rs4671601 | *[LOC339807]* | C | T | 0.806 | 0.966 | 0.038 |  |  |
| 2 | 102926362 | rs12470864 | *IL1RL2-[]-IL18R1* | A | G | 0.385 | 0.997 | 0.055 |  |  |
| 2 | 102941311 | rs10865050 | *[IL18R1]* | G | A | 0.866 | 0.996 | 0.122 |  |  |
| 2 | 112269127 | rs13403656 | *BCL2L11--[]--ANAPC1* | A | T | 0.173 | 0.827 | 0.044 |  |  |
| 2 | 112388538 | rs4848612 | *BCL2L11--[]--ANAPC1* | A | G | 0.773 | 0.996 | 0.037 |  |  |
| 2 | 113590467 | rs1143633 | *[IL1B]* | C | T | 0.644 | 0.96 | 0.033 |  |  |
| 2 | 143831599 | rs74847330 | *KYNU-[]-ARHGAP15* | A | G | 0.879 | 0.942 | 0.047 |  |  |
| 2 | 198950240 | rs1064213 | *[PLCL1]* | G | A | 0.517 | 1 | 0.034 |  |  |
| 2 | 228707862 | rs13384448 | *CCL20-[]-DAW1* | T | C | 0.755 | 0.986 | 0.041 |  |  |
| 2 | 234115629 | rs1057258 | *[INPP5D]* | C | T | 0.815 | 0.969 | 0.044 |  |  |
| 3 | 33069091 | rs6776757 | *[GLB1]* | G | A | 0.507 | 0.995 | 0.032 |  |  |
| 3 | 72394852 | rs61192126 | *LINC00870--[]-RYBP* | T | C | 0.702 | 0.946 | 0.036 |  |  |
| 3 | 101242751 | rs13088318 | *FAM172BP-[]-TRMT10C* | A | G | 0.663 | 0.992 | 0.031 |  |  |
| 3 | 121652141 | rs75557865 | *[SLC15A2]* | G | A | 0.528 | 1 | 0.029 |  |  |
| 3 | 141321836 | rs10663129 | *[RASA2]* | ACT | A | 0.337 | 0.993 | 0.041 |  |  |
| 3 | 187633268 | rs519973 | *BCL6--[]--LPP-AS2* | A | G | 0.339 | 0.987 | 0.033 |  |  |
| 3 | 187793833 | rs2030030 | *BCL6--[]-LPP-AS2* | T | C | 0.844 | 0.979 | 0.040 |  |  |
| 3 | 188133336 | rs60946162 | *[LPP]* | T | C | 0.458 | 0.96 | 0.040 |  |  |
| 3 | 188402586 | rs17607589 | *[LPP]* | C | T | 0.832 | 0.998 | 0.052 |  |  |
| 3 | 196372546 | rs80064395 | *FBXO45-[]-CEP19* | C | T | 0.932 | 0.826 | 0.068 |  |  |
| 4 | 4775401 | rs76991385 | *STX18--[]-MSX1* | G | A | 0.36 | 0.979 | 0.039 |  | rs10033073 |
| 4 | 38798648 | rs5743618 | *[TLR1]* | C | A | 0.748 | 0.935 | 0.095 |  |  |
| 4 | 103593898 | rs227275 | *[MANBA]* | C | A | 0.534 | 0.984 | 0.033 |  |  |
| 4 | 123316076 | rs4145717 | *[ADAD1]* | T | G | 0.333 | 1 | 0.057 |  |  |
| 4 | 123454110 | rs150254607 | *IL2-[]-IL21* | ATAT | A | 0.075 | 0.988 | 0.075 |  |  |
| 5 | 14690667 | rs78486128 | *[FAM105A]* | G | A | 0.935 | 0.815 | 0.069 | rs16903574 |  |
| 5 | 35862841 | rs7717955 | *[IL7R]* | C | T | 0.724 | 0.997 | 0.070 |  |  |
| 5 | 40492655 | rs7714574 | *DAB2---[]--PTGER4* | T | C | 0.604 | 0.989 | 0.031 |  |  |
| 5 | 110159879 | rs1814576 | *SLC25A46-[]--TSLP* | C | T | 0.065 | 0.95 | 0.114 |  |  |
| 5 | 110166083 | rs6869502 | *SLC25A46-[]--TSLP* | T | A | 0.149 | 0.995 | 0.076 |  |  |
| 5 | 110401872 | rs1837253 | *SLC25A46--[]-TSLP* | C | T | 0.742 | 1 | 0.068 |  |  |
| 5 | 110470137 | rs6594499 | *WDR36-[]-CAMK4* | C | A | 0.515 | 0.985 | 0.072 |  |  |
| 5 | 118684297 | rs250308 | *[TNFAIP8]* | T | C | 0.389 | 0.999 | 0.031 |  |  |
| 5 | 131799626 | rs3749833 | *[C5orf56]* | C | T | 0.258 | 0.996 | 0.038 |  |  |
| 5 | 131989136 | rs3091307 | *RAD50-[]-IL13* | G | A | 0.197 | 0.966 | 0.060 |  |  |
| 5 | 131996500 | rs848 | *[IL13]* | A | C | 0.184 | 0.982 | 0.066 |  |  |
| 5 | 140925362 | rs740474 | *[DIAPH1]* | C | T | 0.398 | 0.999 | 0.033 |  |  |
| 5 | 141494934 | rs10068717 | *[NDFIP1]* | T | C | 0.615 | 1 | 0.041 |  |  |
| 5 | 159909345 | rs2910162 | *MIR3142-[]-MIR146A* | G | A | 0.674 | 0.989 | 0.032 |  |  |
| 5 | 176782218 | rs13153019 | *LMAN2-[]-RGS14* | C | T | 0.25 | 0.956 | 0.034 |  |  |
| 6 | 29893575 | rs9259819 | *[HLA-J]* | G | T | 0.519 | 0.927 | 0.035 |  |  |
| 6 | 31323012 | rs2854001 | *[HLA-B]* | A | G | 0.24 | 0.986 | 0.057 |  |  |
| 6 | 31351664 | rs114333358 | *HLA-B-[]-MICA* | G | A | 0.422 | 0.998 | 0.034 |  | rs2507978 |
| 6 | 31574525 | rs149130795 | *NCR3-[]-AIF1* | C | T | 0.939 | 0.998 | 0.093 |  | rs28895016 |
| 6 | 32626403 | rs113453523 | *HLA-DQA1-[]-HLA-DQB1* | A | G | 0.761 | 0.971 | 0.096 |  | rs34004019 |
| 6 | 33046752 | rs114305667 | *[HLA-DPA1]* | G | C | 0.883 | 0.986 | 0.062 |  | rs3097670 |
| 6 | 33647058 | rs10947428 | *[ITPR3]* | C | T | 0.215 | 0.964 | 0.045 |  |  |
| 6 | 90987512 | rs2134814 | *[BACH2]* | C | G | 0.645 | 0.996 | 0.045 |  |  |
| 6 | 106667535 | rs9372120 | *[ATG5]* | G | T | 0.215 | 0.998 | 0.041 |  |  |
| 6 | 128294709 | rs35469349 | *[PTPRK]* | A | T | 0.278 | 0.941 | 0.036 |  |  |
| 6 | 138195151 | rs5029937 | *[TNFAIP3]* | G | T | 0.969 | 0.998 | 0.078 |  |  |
| 6 | 157419508 | rs9383820 | *[ARID1B]* | C | T | 0.792 | 1 | 0.036 |  |  |
| 6 | 167390671 | rs72033857 | *RNASET2-[]-MIR3939* | C | CCTTT | 0.109 | 0.96 | 0.055 |  |  |
| 7 | 20376018 | rs10486391 | *[ITGB8]* | A | G | 0.584 | 1 | 0.030 |  |  |
| 7 | 20560996 | rs6461503 | *ITGB8--[]-ABCB5* | T | C | 0.482 | 1 | 0.038 |  |  |
| 7 | 28156887 | rs6977955 | *[JAZF1]* | T | C | 0.202 | 0.996 | 0.045 |  |  |
| 7 | 50253897 | rs17664743 | *C7orf72-[]-IKZF1* | A | G | 0.201 | 0.985 | 0.041 |  |  |
| 7 | 77018542 | rs4296977 | *[GSAP]* | C | T | 0.14 | 0.998 | 0.054 |  |  |
| 8 | 81292599 | rs7824394 | *MIR5708--[]--ZBTB10* | A | C | 0.358 | 0.998 | 0.049 |  |  |
| 8 | 128814091 | rs6990534 | *[MYC]* | A | G | 0.296 | 0.993 | 0.041 |  |  |
| 9 | 5064193 | rs16922576 | *[JAK2]* | C | T | 0.278 | 0.978 | 0.035 |  |  |
| 9 | 6051399 | rs343478 | *RANBP6-[]--IL33* | G | A | 0.544 | 0.977 | 0.032 |  |  |
| 9 | 6208030 | rs144829310 | *RANBP6--[]-IL33* | T | G | 0.17 | 0.999 | 0.086 |  |  |
| 9 | 123650534 | rs10760123 | *PHF19-[]-TRAF1* | T | G | 0.397 | 0.928 | 0.031 |  |  |
| 9 | 131613191 | rs12551834 | *C9orf114-[]-LRRC8A* | G | A | 0.912 | 0.881 | 0.056 |  |  |
| 10 | 6074451 | rs4747846 | *[IL2RA]* | C | G | 0.479 | 0.972 | 0.035 |  |  |
| 10 | 6094697 | rs61839660 | *[IL2RA]* | T | C | 0.1 | 0.982 | 0.077 |  |  |
| 10 | 8605553 | rs11255753 | *GATA3--[]SFTA1P* | T | G | 0.278 | 1 | 0.040 |  |  |
| 10 | 8841669 | rs2025758 | *GATA3---[]---SFTA1P* | T | C | 0.553 | 1 | 0.040 |  |  |
| 10 | 8936162 | rs11255968 | *GATA3---[]---SFTA1P* | C | T | 0.971 | 0.987 | 0.086 |  |  |
| 10 | 9032555 | rs72782676 | *GATA3---[]---SFTA1P* | C | G | 0.993 | 0.839 | 0.265 |  |  |
| 10 | 9049253 | rs12413578 | *GATA3---[]---SFTA1P* | C | T | 0.894 | 0.929 | 0.091 |  |  |
| 10 | 9064361 | rs1444789 | *GATA3---[]---SFTA1P* | C | T | 0.189 | 0.981 | 0.064 |  |  |
| 10 | 64382359 | rs2893907 | *[ZNF365]* | C | A | 0.533 | 0.985 | 0.031 |  |  |
| 10 | 104225832 | rs10883723 | *C10orf95-[]-ACTR1A* | C | T | 0.355 | 0.996 | 0.030 |  |  |
| 11 | 65551957 | rs479844 | *AP5B1-[]-OVOL1* | G | A | 0.556 | 1 | 0.037 |  |  |
| 11 | 76293758 | rs7936323 | *WNT11--[]-LRRC32* | A | G | 0.485 | 0.982 | 0.084 |  |  |
| 11 | 76299431 | rs55646091 | *WNT11--[]-LRRC32* | A | G | 0.053 | 0.922 | 0.165 |  |  |
| 11 | 76343428 | rs11236814 | *WNT11--[]-LRRC32* | A | T | 0.91 | 0.875 | 0.066 |  |  |
| 11 | 95425526 | rs59593577 | *SESN3--[]-FAM76B* | C | T | 0.874 | 0.806 | 0.052 |  |  |
| 11 | 111470567 | rs7130753 | *LAYN-[]-SIK2* | C | T | 0.729 | 0.992 | 0.044 |  |  |
| 11 | 118743286 | rs12365699 | *DDX6-[]-CXCR5* | G | A | 0.841 | 1 | 0.059 |  |  |
| 11 | 128158189 | rs56129466 | *KIRREL3-AS3---[]--ETS1* | A | G | 0.794 | 0.972 | 0.046 |  |  |
| 12 | 48196982 | rs55726902 | *[HDAC7]* | G | A | 0.765 | 0.977 | 0.050 |  |  |
| 12 | 50345671 | rs11169225 | *[AQP2]* | A | T | 0.198 | 0.992 | 0.044 |  |  |
| 12 | 56401085 | rs10876864 | *SUOX-[]-IKZF4* | G | A | 0.419 | 1 | 0.046 |  |  |
| 12 | 57489709 | rs1059513 | *[STAT6]* | T | C | 0.893 | 1 | 0.081 |  |  |
| 12 | 111932800 | rs7137828 | *[ATXN2]* | T | C | 0.521 | 0.999 | 0.032 |  |  |
| 12 | 121363724 | rs6489785 | *SPPL3-[]-HNF1A-AS1* | T | C | 0.392 | 0.986 | 0.042 |  |  |
| 12 | 123742692 | rs63406760 | *C12orf65-[]-CDK2AP1* | T | TG | 0.786 | 0.991 | 0.046 |  |  |
| 13 | 41173408 | rs4943794 | *[FOXO1]* | C | G | 0.213 | 1 | 0.042 |  |  |
| 13 | 73627275 | rs9573092 | *PIBF1-[]-KLF5* | A | G | 0.645 | 0.987 | 0.030 |  |  |
| 14 | 35761675 | rs1048990 | *[PSMA6]* | G | C | 0.176 | 0.992 | 0.038 |  |  |
| 14 | 38097001 | rs111914382 | *FOXA1-[]--TTC6* | TG | T | 0.24 | 0.991 | 0.035 |  |  |
| 14 | 68754417 | rs2104047 | *[RAD51B]* | T | C | 0.279 | 0.998 | 0.041 |  |  |
| 14 | 75968608 | rs9323612 | *JDP2-[]-BATF* | A | G | 0.67 | 0.992 | 0.031 |  |  |
| 14 | 103235012 | rs9989163 | *RCOR1-[]-TRAF3* | A | G | 0.467 | 0.986 | 0.029 |  |  |
| 15 | 41782684 | rs12440045 | *RTF1-[]-ITPKA* | C | A | 0.52 | 0.94 | 0.032 |  |  |
| 15 | 61068347 | rs10519067 | *[RORA]* | G | A | 0.874 | 0.997 | 0.054 |  |  |
| 15 | 67448363 | rs56375023 | *[SMAD3]* | A | G | 0.246 | 0.994 | 0.070 |  |  |
| 15 | 91045408 | rs3540 | *[IQGAP1]* | G | A | 0.682 | 0.999 | 0.035 |  |  |
| 16 | 11277358 | rs11644510 | *CLEC16A-[]-RMI2* | C | T | 0.638 | 0.952 | 0.070 |  |  |
| 16 | 11491007 | rs12596613 | *RMI2-[]--LITAF* | C | G | 0.66 | 0.937 | 0.031 |  |  |
| 17 | 4521473 | rs71368508 | *SMTNL2-[]-ALOX15* | C | A | 0.979 | 0.845 | 0.117 |  |  |
| 17 | 38069076 | rs921650 | *[GSDMB]* | A | G | 0.478 | 1 | 0.057 |  |  |
| 17 | 38149033 | rs11652139 | *[PSMD3]* | A | G | 0.563 | 0.98 | 0.050 |  |  |
| 17 | 38604546 | rs10305290 | *CCR7-[]-SMARCE1* | T | C | 0.014 | 0.904 | 0.231 | rs112401631 |  |
| 17 | 38770641 | rs11464691 | *CCR7-[]-SMARCE1* | TA | T | 0.632 | 0.995 | 0.051 |  |  |
| 17 | 40414862 | rs7207591 | *[STAT5B]* | A | G | 0.754 | 0.869 | 0.037 |  |  |
| 17 | 43430696 | rs116053081 | *MAP3K14-[]-ARHGAP27* | G | A | 0.353 | 1 | 0.031 |  | rs7214661 |
| 17 | 47398070 | rs9889262 | *[ZNF652]* | A | T | 0.371 | 0.997 | 0.042 |  |  |
| 18 | 52336175 | rs4801001 | *DYNAP-[]--RAB27B* | T | C | 0.469 | 0.922 | 0.031 |  |  |
| 18 | 60009814 | rs4574025 | *[TNFRSF11A]* | T | C | 0.545 | 0.947 | 0.030 |  |  |
| 19 | 33721455 | rs10414065 | *SLC7A10-[]-CEBPA* | C | T | 0.936 | 0.851 | 0.093 |  |  |
| 20 | 50157837 | rs3787184 | *[NFATC2]* | A | G | 0.82 | 0.88 | 0.048 |  |  |
| 20 | 52208356 | rs2766678 | *[ZNF217]* | G | A | 0.218 | 0.941 | 0.055 |  |  |
| 20 | 62322699 | rs6011033 | *[RTEL1]* | G | A | 0.772 | 0.956 | 0.046 |  |  |
| 21 | 36467830 | rs73205303 | *[RUNX1]* | A | G | 0.158 | 0.997 | 0.043 |  |  |
| 21 | 44846426 | rs76081789 | *[SIK1]* | T | C | 0.944 | 0.987 | 0.063 |  |  |
| 22 | 41816652 | rs5758343 | *TEF-[]-TOB2* | A | T | 0.204 | 0.997 | 0.047 |  |  |

* Imputation quality measure

† Beta from regression Ferreira et al ([2](#_ENREF_2))

‡ Duplicate SNPs as in dbSNP records ([3](#_ENREF_3))

### **Table S2**. Power calculations for associations with individual latent classes based on ALSPAC data.

|  |  |  |  | **Risk allele frequency (RAF)** | | | |
| --- | --- | --- | --- | --- | --- | --- | --- |
| **Allergic diseases latent classes** | **N cases** | **Prev.*** | **RRR**† | 0.05 | 0.15 | 0.25 | 0.45 |
| Atopic March | 173 | 0.031 | 1.10 | 0.001 | 0.002 | 0.002 | 0.003 |
|  |  |  | 1.30 | 0.006 | 0.030 | 0.058 | 0.082 |
|  |  |  | 1.50 | 0.027 | 0.159 | 0.282 | 0.351 |
| Persistent eczema and wheeze | 154 | 0.027 | 1.10 | 0.001 | 0.001 | 0.002 | 0.003 |
|  |  |  | 1.30 | 0.005 | 0.024 | 0.045 | 0.064 |
|  |  |  | 1.50 | 0.021 | 0.123 | 0.223 | 0.281 |
| Persistent eczema later-onset rhinitis | 279 | 0.047 | 1.10 | 0.001 | 0.003 | 0.005 | 0.007 |
|  |  |  | 1.30 | 0.014 | 0.082 | 0.158 | 0.221 |
|  |  |  | 1.50 | 0.073 | 0.398 | 0.611 | 0.699 |
| Persistent wheeze later-onset rhinitis | 328 | 0.057 | 1.10 | 0.001 | 0.004 | 0.006 | 0.009 |
|  |  |  | 1.30 | 0.019 | 0.116 | 0.221 | 0.302 |
|  |  |  | 1.50 | 0.103 | 0.517 | 0.736 | **0.813** |
| Transient wheeze | 491 | 0.077 | 1.10 | 0.002 | 0.006 | 0.011 | 0.017 |
|  |  |  | 1.30 | 0.041 | 0.252 | 0.440 | 0.558 |
|  |  |  | 1.50 | 0.229 | **0.803** | **0.941** | **0.969** |
| Eczema only | 971 | 0.153 | 1.10 | 0.005 | 0.022 | 0.044 | 0.070 |
|  |  |  | 1.30 | 0.185 | 0.747 | **0.920** | **0.964** |
|  |  |  | 1.50 | 0.725 | **0.998** | **1.000** | **1.000** |
| Rhinitis only | 602 | 0.096 | 1.10 | 0.002 | 0.009 | 0.016 | 0.025 |
|  |  |  | 1.30 | 0.063 | 0.371 | 0.596 | 0.716 |
|  |  |  | 1.50 | 0.343 | **0.915** | **0.985** | **0.993** |

* Prevalence of each class

† Relative risk ratio (Multinomial odds ratio)

Highlighted values indicate where statistical power > 0.80 (which is generally considered a standard for power adequacy).

### **Table S3.** Associations between genetic scores and profile latent classes (weighted by membership probabilities) before and after excluding SNPs rs61816761 (*FLG*) and rs921650 (*GSDMB*) in ALSPAC cohort.

|  | **Multinomial Odds Ratio (95% CI)** | |
| --- | --- | --- |
| **Allergic diseases latent classes** | **Stand. Score** | **Stand. Score excluding *FLG* and *GSDMB*** |
| Atopic March | 2.021 (1.731,2.360) | 1.931 (1.654,2.254) |
| Persistent eczema and wheeze | 1.397 (1.200,1.626) | 1.353 (1.163,1.575) |
| Persistent eczema later-onset rhinitis | 1.543 (1.370,1.739) | 1.525 (1.354,1.717) |
| Persistent wheeze later-onset rhinitis | 1.420 (1.269,1.589) | 1.395 (1.247,1.560) |
| Transient wheeze | 1.108 (1.009,1.216) | 1.089 (0.992,1.195) |
| Eczema only | 1.177 (1.093,1.267) | 1.171 (1.088,1.260) |
| Rhinitis only | 1.196 (1.099,1.301) | 1.202 (1.105,1.307) |
| **P-value for differential association*** | 3.28E-13 | 9.56E-12 |
| **P-value for any association†** | 3.19E-30 | 2.43E-27 |

Bonferroni corrected significance threshold is 0.000370 (0.05/135)

* Heterogeneity p value from chi-square test excluding the baseline class of ‘no disease’

† Heterogeneity p value from chi-square test including the baseline class of ‘no disease’ (Multinomial Odds Ratio =1)

### **Table S4** Pairwise tests among disease latent classes from associations between genetic scores and profile latent classes (Table S3) in ALSPAC.

|  |  | **Atopic March** | **Persistent eczema and wheeze** | **Persistent eczema LO rhinitis** | **Persistent wheeze LO rhinitis** | **Transient wheeze** | **Eczema only** | **Rhinitis only** |
| --- | --- | --- | --- | --- | --- | --- | --- | --- |
|  |  | standard genetic score | | | | | | |
| **Atopic March** | standard genetic score excluding *FLG* and *GSDMB* |  | 5.51E-04 | 4.64E-03 | 1.56E-04 | 1.00E-11 | 8.10E-11 | 9.68E-10 |
| **Persistent eczema and wheeze** |  | 8.99E-04 |  | 0.29 | 0.86 | 7.85E-03 | 0.04 | 0.07 |
| **Persistent eczema LO rhinitis** |  | 0.013 | 0.21 |  | 0.29 | 5.06E-06 | 4.44E-05 | 2.52E-04 |
| **Persistent wheeze LO rhinitis** |  | 5.03E-04 | 0.74 | 0.26 |  | 3.77E-04 | 2.98E-03 | 0.010 |
| **Transient wheeze** |  | 8.73E-11 | 0.012 | 3.51E-06 | 3.72E-04 |  | 0.28 | 0.19 |
| **Eczema only** |  | 1.81E-09 | 0.08 | 6.86E-05 | 5.45E-03 | 0.19 |  | 0.75 |
| **Rhinitis only** |  | 3.12E-08 | 0.16 | 6.10E-04 | 2.44E-02 | 0.09 | 0.61 |  |

### **Table S5.** Individual SNP associations with profile latent classes in ALSPAC (analysis weighted by class membership probabilities)

|  | **Multinomial Odds Ratio (95% CI) for each AD latent class** | | | | | | |  |  |
| --- | --- | --- | --- | --- | --- | --- | --- | --- | --- |
| **SNP** | **Atopic March** | **Persistent eczema and wheeze** | **Persistent eczema later-onset rhinitis** | **Persistent wheeze later-onset rhinitis** | **Transient wheeze** | **Eczema only** | **Rhinitis only** | **P-value differential association*** | **P-value any association†** |
| **Nominal SNPs** |  |  |  |  |  |  |  |  |  |
| rs921650 (*GSDMB*) | 1.327 (1.068,1.648) | 1.289 (1.041,1.596) | 0.952 (0.805,1.125) | 1.336 (1.141,1.565) | 1.246 (1.093,1.420) | 0.953 (0.860,1.057) | 0.978 (0.869,1.101) | 1.42E-05 | 5.32E-06 |
| rs61816761 (*FLG*) | 3.254 (1.830,5.789) | 1.934 (0.975,3.838) | 2.440 (1.479,4.024) | 0.913 (0.464,1.796) | 1.019 (0.595,1.744) | 1.680 (1.165,2.422) | 0.981 (0.599,1.606) | 0.0019 | 3.60E-05 |
| rs11652139 | 1.446 (1.153,1.812) | 1.131 (0.910,1.405) | 1.086 (0.917,1.286) | 1.267 (1.078,1.490) | 1.190 (1.042,1.360) | 0.961 (0.866,1.067) | 1.048 (0.930,1.181) | 0.003 | 0.0006 |
| rs479844 | 1.133 (0.909,1.414) | 1.207 (0.970,1.503) | 1.233 (1.038,1.463) | 0.951 (0.811,1.115) | 1.057 (0.925,1.206) | 1.132 (1.019,1.257) | 0.939 (0.834,1.058) | 0.0336 | 0.0189 |
| rs6990534 | 1.350 (1.076,1.693) | 0.949 (0.748,1.203) | 1.069 (0.892,1.282) | 1.037 (0.873,1.232) | 0.944 (0.817,1.092) | 0.947 (0.844,1.062) | 1.132 (0.997,1.286) | 0.0382 | 0.0541 |
| rs5743618 | 1.491 (1.126,1.974) | 1.033 (0.802,1.329) | 1.076 (0.882,1.313) | 1.179 (0.973,1.427) | 0.950 (0.816,1.105) | 1.020 (0.904,1.152) | 1.188 (1.030,1.372) | 0.0487 | 0.0253 |
| **Disease-Specific SNPs** | |  |  |  |  |  |  |  |  |
| rs115288876 (*LOC105371442*) | 1.774 (1.134,2.775) | 1.163 (0.695,1.946) | 1.024 (0.671,1.563) | 0.733 (0.466,1.151) | 0.829 (0.580,1.184) | 1.138 (0.883,1.466) | 1.059 (0.788,1.425) | 0.0902 | 0.1218 |
| rs12470864 (*IL1RL1*) | 0.867 (0.690,1.091) | 1.059 (0.849,1.320) | 1.011 (0.850,1.202) | 1.053 (0.895,1.239) | 1.089 (0.952,1.246) | 0.960 (0.862,1.069) | 1.096 (0.971,1.238) | 0.3626 | 0.4266 |
| rs61839660 (*IL2RA*) | 1.033 (0.724,1.474) | 1.032 (0.727,1.464) | 1.222 (0.944,1.582) | 0.978 (0.752,1.273) | 0.953 (0.765,1.188) | 1.076 (0.910,1.272) | 0.903 (0.737,1.106) | 0.5685 | 0.6706 |
| rs6594499  (WDR36 and CAMK4) | 1.089 (0.877,1.353) | 1.055 (0.852,1.307) | 1.082 (0.916,1.279) | 1.183 (1.010,1.385) | 1.032 (0.905,1.176) | 1.094 (0.986,1.213) | 1.051 (0.934,1.183) | 0.8866 | 0.4412 |
| **Other SNPs** | |  |  |  |  |  |  |  |  |
| rs2104047 | 1.226 (0.974,1.544) | 1.258 (1.004,1.577) | 1.150 (0.961,1.376) | 1.100 (0.927,1.305) | 0.928 (0.802,1.075) | 0.980 (0.874,1.099) | 0.978 (0.858,1.115) | 0.0714 | 0.0961 |
| rs7512552 | 1.179 (0.950,1.463) | 1.142 (0.923,1.412) | 1.112 (0.942,1.313) | 0.952 (0.815,1.113) | 0.874 (0.768,0.995) | 1.005 (0.907,1.113) | 1.029 (0.915,1.156) | 0.0852 | 0.1329 |
| rs6776757 | 1.122 (0.904,1.393) | 1.084 (0.876,1.341) | 0.942 (0.798,1.113) | 1.159 (0.990,1.357) | 1.092 (0.959,1.244) | 0.945 (0.852,1.047) | 0.949 (0.843,1.067) | 0.1087 | 0.1645 |
| rs7936323 | 1.412 (1.137,1.752) | 0.976 (0.790,1.206) | 1.029 (0.872,1.213) | 0.986 (0.843,1.152) | 1.043 (0.917,1.187) | 1.060 (0.957,1.174) | 1.033 (0.919,1.161) | 0.174 | 0.1392 |
| rs7824394 | 1.279 (1.028,1.591) | 1.218 (0.981,1.512) | 1.025 (0.863,1.217) | 1.074 (0.914,1.262) | 1.028 (0.898,1.176) | 0.979 (0.879,1.089) | 0.980 (0.867,1.107) | 0.1951 | 0.2317 |
| rs343478 | 1.105 (0.886,1.378) | 1.018 (0.820,1.264) | 0.865 (0.731,1.023) | 1.112 (0.947,1.305) | 0.935 (0.819,1.067) | 0.987 (0.889,1.096) | 0.908 (0.806,1.023) | 0.1987 | 0.2267 |
| rs1059513 | 1.432 (0.969,2.118) | 1.453 (0.986,2.140) | 1.223 (0.921,1.622) | 1.196 (0.918,1.559) | 1.097 (0.887,1.357) | 1.190 (1.002,1.414) | 0.946 (0.787,1.137) | 0.1995 | 0.0679 |
| rs3091307 | 1.113 (0.847,1.463) | 1.239 (0.954,1.609) | 1.339 (1.094,1.637) | 1.112 (0.911,1.356) | 1.093 (0.926,1.290) | 1.032 (0.903,1.179) | 0.986 (0.845,1.150) | 0.2158 | 0.1124 |
| rs2766678 | 0.959 (0.732,1.258) | 0.961 (0.736,1.254) | 1.065 (0.869,1.305) | 0.848 (0.692,1.039) | 0.885 (0.750,1.045) | 1.086 (0.957,1.231) | 0.962 (0.830,1.115) | 0.2422 | 0.3254 |
| rs12365699 | 1.241 (0.906,1.700) | 1.078 (0.802,1.447) | 1.190 (0.938,1.510) | 0.931 (0.757,1.146) | 0.935 (0.787,1.110) | 0.951 (0.829,1.091) | 1.109 (0.941,1.307) | 0.2447 | 0.3329 |
| rs4574025 | 1.383 (1.103,1.735) | 1.101 (0.885,1.370) | 1.022 (0.862,1.212) | 1.167 (0.993,1.373) | 1.028 (0.900,1.175) | 1.055 (0.949,1.172) | 1.025 (0.908,1.156) | 0.2456 | 0.1254 |
| rs12440045 | 1.249 (0.997,1.564) | 1.015 (0.815,1.264) | 0.964 (0.812,1.144) | 1.109 (0.943,1.305) | 0.973 (0.851,1.112) | 1.028 (0.925,1.144) | 0.940 (0.833,1.062) | 0.2825 | 0.3761 |
| rs7207591 | 0.967 (0.741,1.262) | 1.127 (0.859,1.479) | 1.279 (1.028,1.592) | 0.996 (0.819,1.210) | 0.921 (0.786,1.080) | 1.033 (0.909,1.175) | 0.992 (0.858,1.148) | 0.2934 | 0.38 |
| rs1057258 | 1.220 (0.900,1.654) | 0.872 (0.664,1.145) | 1.124 (0.894,1.412) | 0.959 (0.780,1.179) | 0.974 (0.821,1.157) | 0.883 (0.773,1.009) | 0.973 (0.833,1.137) | 0.3158 | 0.3449 |
| rs114333358 | 0.854 (0.685,1.066) | 0.959 (0.773,1.189) | 1.015 (0.859,1.200) | 1.143 (0.977,1.337) | 0.996 (0.874,1.135) | 0.995 (0.897,1.103) | 1.086 (0.965,1.222) | 0.3252 | 0.3984 |
| rs2228145 | 1.350 (1.089,1.673) | 1.112 (0.899,1.376) | 1.108 (0.938,1.309) | 1.013 (0.865,1.187) | 1.157 (1.015,1.317) | 1.050 (0.946,1.164) | 1.057 (0.939,1.190) | 0.3294 | 0.0836 |
| rs848 | 1.423 (1.097,1.846) | 1.354 (1.045,1.755) | 1.264 (1.027,1.555) | 1.130 (0.923,1.382) | 1.118 (0.945,1.322) | 1.093 (0.956,1.249) | 1.087 (0.933,1.267) | 0.3398 | 0.0264 |
| rs4943794 | 1.210 (0.941,1.555) | 1.193 (0.931,1.530) | 1.199 (0.987,1.456) | 1.070 (0.886,1.292) | 1.026 (0.876,1.202) | 1.002 (0.884,1.136) | 0.969 (0.838,1.120) | 0.3604 | 0.3535 |
| rs4848612 | 1.370 (1.039,1.807) | 1.095 (0.848,1.414) | 1.109 (0.908,1.355) | 1.010 (0.840,1.215) | 0.967 (0.831,1.126) | 1.058 (0.936,1.195) | 1.134 (0.984,1.307) | 0.3607 | 0.2395 |
| rs144829310 | 1.201 (0.913,1.579) | 1.224 (0.936,1.600) | 1.157 (0.935,1.433) | 1.229 (1.008,1.498) | 1.097 (0.925,1.300) | 1.001 (0.872,1.149) | 0.997 (0.852,1.168) | 0.3681 | 0.2347 |
| rs3540 | 1.123 (0.889,1.417) | 1.254 (0.992,1.586) | 1.021 (0.856,1.218) | 1.199 (1.010,1.423) | 1.069 (0.930,1.228) | 0.999 (0.896,1.114) | 1.103 (0.972,1.251) | 0.3789 | 0.192 |
| rs10174949 | 1.294 (1.009,1.660) | 1.239 (0.972,1.579) | 1.051 (0.876,1.262) | 1.042 (0.877,1.237) | 0.967 (0.841,1.114) | 1.084 (0.968,1.214) | 1.075 (0.944,1.224) | 0.3844 | 0.2292 |
| rs2854001 | 1.241 (0.977,1.576) | 0.956 (0.745,1.228) | 1.110 (0.919,1.341) | 0.936 (0.778,1.127) | 0.928 (0.795,1.082) | 0.999 (0.886,1.126) | 1.006 (0.878,1.154) | 0.386 | 0.4998 |
| rs1102705 | 1.050 (0.732,1.507) | 0.887 (0.608,1.294) | 1.299 (1.002,1.684) | 1.137 (0.880,1.468) | 0.944 (0.753,1.182) | 0.966 (0.809,1.153) | 1.065 (0.875,1.296) | 0.407 | 0.4814 |
| rs17664743 | 0.793 (0.595,1.056) | 1.135 (0.880,1.465) | 1.057 (0.863,1.295) | 0.898 (0.735,1.097) | 0.909 (0.770,1.072) | 0.990 (0.871,1.125) | 0.930 (0.802,1.079) | 0.4267 | 0.4384 |
| rs9323612 | 1.103 (0.873,1.393) | 1.116 (0.886,1.406) | 1.103 (0.922,1.321) | 1.144 (0.964,1.357) | 0.951 (0.829,1.092) | 1.124 (1.005,1.256) | 1.016 (0.896,1.152) | 0.4273 | 0.2681 |
| rs1048990 | 1.322 (1.014,1.724) | 1.077 (0.817,1.419) | 1.150 (0.930,1.422) | 1.051 (0.856,1.291) | 0.948 (0.796,1.130) | 1.102 (0.965,1.260) | 1.026 (0.878,1.198) | 0.4403 | 0.3366 |
| rs13403656 | 1.159 (0.858,1.567) | 1.138 (0.844,1.534) | 1.056 (0.832,1.341) | 1.001 (0.797,1.258) | 0.966 (0.798,1.170) | 1.164 (1.008,1.346) | 0.957 (0.804,1.138) | 0.4612 | 0.4454 |
| rs2070901 | 1.235 (0.975,1.564) | 0.928 (0.725,1.188) | 1.110 (0.922,1.337) | 1.090 (0.914,1.300) | 0.988 (0.852,1.147) | 0.977 (0.868,1.099) | 1.039 (0.909,1.188) | 0.4663 | 0.5267 |
| rs2025758 | 1.150 (0.922,1.436) | 1.110 (0.892,1.379) | 1.089 (0.919,1.291) | 1.165 (0.991,1.368) | 1.101 (0.964,1.258) | 0.982 (0.884,1.090) | 1.027 (0.911,1.158) | 0.4671 | 0.3427 |
| rs55646091 | 1.283 (0.800,2.058) | 0.900 (0.527,1.537) | 1.512 (1.071,2.134) | 0.941 (0.639,1.386) | 1.136 (0.842,1.533) | 1.147 (0.904,1.454) | 1.037 (0.783,1.372) | 0.4751 | 0.3506 |
| rs10305290 | 2.207 (1.073,4.540) | 1.147 (0.454,2.897) | 0.997 (0.462,2.150) | 0.897 (0.421,1.913) | 1.070 (0.596,1.921) | 1.081 (0.681,1.717) | 1.502 (0.939,2.404) | 0.4901 | 0.4087 |
| rs7714574 | 1.124 (0.898,1.407) | 1.060 (0.852,1.321) | 0.984 (0.830,1.167) | 1.110 (0.943,1.306) | 1.117 (0.976,1.278) | 0.968 (0.871,1.076) | 1.061 (0.940,1.198) | 0.5029 | 0.4779 |
| rs6461503 | 1.225 (0.985,1.525) | 1.027 (0.828,1.272) | 0.971 (0.821,1.149) | 1.004 (0.857,1.177) | 0.990 (0.868,1.129) | 0.940 (0.847,1.043) | 1.001 (0.889,1.127) | 0.5031 | 0.612 |
| rs60946162 | 0.917 (0.737,1.142) | 1.025 (0.826,1.270) | 0.985 (0.833,1.165) | 1.080 (0.922,1.265) | 0.898 (0.787,1.025) | 1.018 (0.918,1.130) | 0.942 (0.836,1.061) | 0.5094 | 0.5836 |
| rs11644510 | 1.055 (0.838,1.327) | 0.971 (0.776,1.215) | 1.017 (0.853,1.213) | 0.955 (0.809,1.126) | 1.103 (0.960,1.268) | 0.946 (0.849,1.054) | 1.060 (0.935,1.202) | 0.533 | 0.6424 |
| rs13384448 | 1.097 (0.848,1.419) | 1.053 (0.819,1.353) | 1.130 (0.925,1.379) | 1.040 (0.864,1.251) | 0.941 (0.810,1.093) | 1.136 (1.005,1.285) | 1.089 (0.947,1.252) | 0.5407 | 0.364 |
| rs61192126 | 1.262 (0.981,1.624) | 1.210 (0.946,1.547) | 1.110 (0.919,1.340) | 1.107 (0.926,1.322) | 0.989 (0.856,1.143) | 1.066 (0.950,1.197) | 1.022 (0.896,1.166) | 0.5512 | 0.3693 |
| rs150254607 | 1.406 (0.988,2.001) | 1.163 (0.799,1.692) | 1.094 (0.809,1.478) | 0.892 (0.655,1.215) | 1.000 (0.784,1.277) | 1.090 (0.904,1.316) | 1.012 (0.813,1.262) | 0.5673 | 0.5796 |
| rs11464691 | 1.165 (0.927,1.463) | 1.130 (0.904,1.413) | 0.898 (0.758,1.064) | 0.996 (0.847,1.172) | 0.999 (0.874,1.143) | 1.015 (0.913,1.129) | 0.992 (0.878,1.120) | 0.5734 | 0.6843 |
| rs227275 | 1.107 (0.891,1.374) | 1.070 (0.865,1.324) | 1.040 (0.881,1.228) | 0.997 (0.853,1.166) | 0.956 (0.840,1.088) | 1.002 (0.904,1.110) | 1.110 (0.987,1.249) | 0.6096 | 0.645 |
| rs740474 | 1.005 (0.806,1.253) | 0.965 (0.776,1.200) | 0.882 (0.743,1.048) | 0.887 (0.754,1.043) | 1.014 (0.888,1.158) | 1.019 (0.918,1.132) | 0.954 (0.845,1.076) | 0.6253 | 0.6612 |
| rs6489785 | 0.940 (0.752,1.176) | 1.030 (0.828,1.282) | 0.934 (0.786,1.110) | 1.087 (0.926,1.276) | 0.927 (0.810,1.060) | 0.958 (0.861,1.065) | 1.032 (0.915,1.165) | 0.6358 | 0.717 |
| rs13153019 | 1.166 (0.909,1.495) | 1.040 (0.809,1.336) | 0.985 (0.808,1.201) | 0.922 (0.762,1.115) | 0.978 (0.837,1.142) | 0.949 (0.838,1.073) | 1.062 (0.925,1.219) | 0.6364 | 0.7435 |
| rs3787184 | 1.239 (0.901,1.702) | 0.911 (0.684,1.214) | 1.075 (0.850,1.359) | 1.062 (0.852,1.324) | 0.951 (0.796,1.136) | 1.016 (0.881,1.172) | 1.111 (0.940,1.314) | 0.6489 | 0.6934 |
| rs10519067 | 1.059 (0.761,1.475) | 1.064 (0.768,1.475) | 1.131 (0.872,1.467) | 1.318 (1.017,1.708) | 1.083 (0.886,1.323) | 0.992 (0.850,1.157) | 1.104 (0.920,1.325) | 0.649 | 0.5207 |
| rs9383820 | 1.141 (0.867,1.501) | 1.191 (0.906,1.566) | 1.071 (0.870,1.317) | 1.175 (0.961,1.436) | 1.016 (0.866,1.192) | 0.988 (0.872,1.121) | 1.009 (0.874,1.166) | 0.6492 | 0.6554 |
| rs3749833 | 1.257 (0.993,1.591) | 0.936 (0.731,1.198) | 0.993 (0.821,1.201) | 1.014 (0.848,1.212) | 1.027 (0.886,1.191) | 0.997 (0.886,1.121) | 0.989 (0.864,1.131) | 0.653 | 0.7486 |
| rs9989163 | 1.220 (0.980,1.519) | 0.916 (0.737,1.138) | 1.003 (0.847,1.188) | 1.074 (0.916,1.260) | 1.017 (0.891,1.160) | 1.023 (0.922,1.136) | 1.018 (0.903,1.147) | 0.6617 | 0.6966 |
| rs4671601 | 1.265 (0.941,1.701) | 1.332 (0.990,1.792) | 1.199 (0.959,1.500) | 1.087 (0.885,1.334) | 1.138 (0.958,1.351) | 1.065 (0.932,1.217) | 1.053 (0.904,1.226) | 0.679 | 0.2467 |
| rs10414065 | 1.227 (0.740,2.034) | 1.349 (0.804,2.262) | 1.266 (0.855,1.876) | 1.136 (0.796,1.622) | 1.018 (0.766,1.351) | 0.958 (0.769,1.193) | 1.183 (0.903,1.550) | 0.6794 | 0.6555 |
| rs71368508 | 0.799 (0.372,1.716) | 1.478 (0.557,3.918) | 1.039 (0.539,2.001) | 1.205 (0.625,2.325) | 0.753 (0.478,1.187) | 0.907 (0.616,1.335) | 1.201 (0.736,1.958) | 0.6808 | 0.7745 |
| rs17607589 | 1.062 (0.795,1.419) | 0.918 (0.698,1.205) | 1.135 (0.904,1.425) | 1.238 (0.992,1.544) | 1.122 (0.940,1.340) | 1.041 (0.908,1.194) | 1.054 (0.900,1.233) | 0.6845 | 0.5011 |
| rs149130795 | 1.152 (0.716,1.853) | 1.119 (0.705,1.777) | 1.119 (0.780,1.605) | 0.919 (0.670,1.260) | 0.899 (0.693,1.166) | 1.166 (0.931,1.461) | 1.039 (0.811,1.331) | 0.7044 | 0.7655 |
| rs1143633 | 1.001 (0.795,1.259) | 1.225 (0.970,1.547) | 1.011 (0.847,1.207) | 1.082 (0.914,1.281) | 1.025 (0.892,1.178) | 0.991 (0.889,1.106) | 0.985 (0.869,1.116) | 0.7084 | 0.7782 |
| rs1444789 | 1.159 (0.889,1.513) | 1.223 (0.944,1.583) | 1.029 (0.833,1.272) | 1.100 (0.904,1.339) | 1.077 (0.914,1.269) | 0.980 (0.858,1.119) | 1.055 (0.909,1.225) | 0.7169 | 0.6516 |
| rs2893907 | 1.178 (0.948,1.465) | 0.924 (0.748,1.143) | 0.963 (0.816,1.137) | 1.036 (0.885,1.212) | 0.969 (0.851,1.103) | 0.980 (0.885,1.086) | 1.006 (0.894,1.131) | 0.7174 | 0.8111 |
| rs760805 | 1.076 (0.864,1.341) | 0.924 (0.745,1.146) | 1.143 (0.964,1.355) | 1.109 (0.944,1.301) | 1.027 (0.900,1.172) | 1.037 (0.934,1.152) | 1.007 (0.894,1.135) | 0.7201 | 0.6683 |
| rs111914382 | 0.958 (0.740,1.241) | 0.921 (0.712,1.191) | 1.014 (0.833,1.233) | 0.933 (0.772,1.127) | 0.951 (0.814,1.112) | 1.070 (0.949,1.207) | 1.051 (0.916,1.207) | 0.7218 | 0.81 |
| rs11255753 | 1.199 (0.949,1.516) | 1.006 (0.793,1.277) | 1.069 (0.889,1.285) | 1.117 (0.940,1.328) | 1.036 (0.896,1.198) | 0.985 (0.878,1.106) | 1.015 (0.889,1.158) | 0.724 | 0.7311 |
| rs10910095 | 1.214 (0.867,1.700) | 0.912 (0.675,1.233) | 0.980 (0.770,1.247) | 1.056 (0.836,1.333) | 1.020 (0.843,1.234) | 1.124 (0.963,1.313) | 1.139 (0.952,1.361) | 0.7305 | 0.6033 |
| rs519973 | 0.988 (0.787,1.240) | 0.960 (0.767,1.202) | 1.028 (0.863,1.223) | 0.975 (0.826,1.150) | 0.905 (0.788,1.040) | 1.009 (0.906,1.124) | 0.907 (0.800,1.028) | 0.7394 | 0.7185 |
| rs116053081 | 0.932 (0.741,1.173) | 1.023 (0.818,1.278) | 0.962 (0.807,1.147) | 1.061 (0.901,1.250) | 1.029 (0.898,1.179) | 0.991 (0.889,1.104) | 1.101 (0.974,1.245) | 0.74 | 0.788 |
| rs5029937 | 1.701 (0.782,3.698) | 0.892 (0.505,1.573) | 1.012 (0.633,1.620) | 0.857 (0.567,1.296) | 1.096 (0.750,1.602) | 0.913 (0.690,1.208) | 1.057 (0.754,1.483) | 0.7418 | 0.8319 |
| rs56375023 | 1.039 (0.814,1.327) | 1.009 (0.792,1.287) | 1.121 (0.932,1.349) | 1.033 (0.865,1.235) | 0.934 (0.803,1.086) | 0.957 (0.850,1.078) | 1.016 (0.888,1.162) | 0.743 | 0.8341 |
| rs10760123 | 1.119 (0.892,1.403) | 1.072 (0.857,1.342) | 1.129 (0.949,1.344) | 1.031 (0.873,1.217) | 0.960 (0.836,1.103) | 1.001 (0.898,1.116) | 1.045 (0.923,1.183) | 0.7563 | 0.7822 |
| rs10876864 | 0.991 (0.796,1.235) | 0.913 (0.734,1.136) | 1.024 (0.865,1.212) | 1.137 (0.970,1.332) | 1.068 (0.936,1.218) | 1.018 (0.917,1.131) | 1.033 (0.917,1.164) | 0.7599 | 0.7463 |
| rs2134814 | 1.004 (0.802,1.256) | 0.896 (0.720,1.114) | 1.042 (0.875,1.239) | 1.077 (0.913,1.270) | 1.008 (0.881,1.154) | 1.049 (0.942,1.168) | 0.962 (0.851,1.086) | 0.7626 | 0.8377 |
| rs11204896 | 1.401 (0.939,2.090) | 1.129 (0.786,1.622) | 1.340 (0.991,1.812) | 1.105 (0.848,1.440) | 1.047 (0.844,1.299) | 1.128 (0.948,1.342) | 1.081 (0.888,1.316) | 0.7703 | 0.3914 |
| rs12596613 | 1.090 (0.859,1.382) | 1.077 (0.852,1.361) | 1.096 (0.912,1.316) | 1.055 (0.888,1.253) | 0.953 (0.828,1.096) | 1.041 (0.930,1.165) | 0.972 (0.856,1.105) | 0.7813 | 0.8351 |
| rs11255968 | 0.971 (0.519,1.815) | 1.156 (0.593,2.253) | 1.327 (0.763,2.308) | 1.055 (0.658,1.691) | 0.872 (0.608,1.252) | 1.159 (0.842,1.595) | 1.249 (0.855,1.822) | 0.7827 | 0.7889 |
| rs10883723 | 0.949 (0.755,1.192) | 0.961 (0.767,1.202) | 0.953 (0.800,1.136) | 1.016 (0.862,1.198) | 0.989 (0.863,1.134) | 0.991 (0.889,1.104) | 1.094 (0.968,1.236) | 0.785 | 0.8636 |
| rs113453523 | 1.069 (0.826,1.383) | 1.242 (0.954,1.618) | 1.196 (0.975,1.466) | 1.209 (0.997,1.466) | 1.046 (0.896,1.220) | 1.078 (0.953,1.219) | 1.124 (0.976,1.296) | 0.7865 | 0.213 |
| rs4090390 | 1.000 (0.769,1.300) | 1.104 (0.857,1.422) | 1.133 (0.930,1.379) | 1.024 (0.847,1.238) | 0.962 (0.820,1.128) | 1.050 (0.928,1.189) | 0.966 (0.836,1.115) | 0.7876 | 0.8422 |
| rs12413578 | 1.176 (0.807,1.714) | 1.292 (0.879,1.897) | 1.230 (0.917,1.651) | 1.015 (0.783,1.316) | 1.155 (0.923,1.445) | 1.097 (0.921,1.305) | 1.001 (0.825,1.215) | 0.7904 | 0.593 |
| rs55726902 | 0.934 (0.725,1.202) | 1.061 (0.821,1.371) | 0.891 (0.735,1.081) | 0.991 (0.823,1.194) | 1.061 (0.907,1.241) | 0.971 (0.860,1.097) | 1.030 (0.895,1.186) | 0.7954 | 0.8728 |
| rs250308 | 1.119 (0.901,1.390) | 1.061 (0.856,1.315) | 1.092 (0.924,1.291) | 0.953 (0.812,1.119) | 0.967 (0.847,1.104) | 1.019 (0.918,1.131) | 1.027 (0.911,1.157) | 0.7974 | 0.8493 |
| rs9889262 | 1.062 (0.852,1.323) | 0.994 (0.799,1.237) | 1.079 (0.911,1.279) | 0.967 (0.822,1.137) | 1.037 (0.908,1.185) | 0.984 (0.885,1.094) | 1.089 (0.966,1.228) | 0.7974 | 0.8227 |
| rs697852 | 1.113 (0.831,1.491) | 1.199 (0.893,1.609) | 1.170 (0.932,1.470) | 1.138 (0.919,1.409) | 1.089 (0.914,1.296) | 1.086 (0.946,1.246) | 0.983 (0.842,1.147) | 0.7984 | 0.5584 |
| rs75557865 | 1.066 (0.861,1.318) | 0.948 (0.770,1.169) | 1.054 (0.895,1.241) | 1.134 (0.971,1.324) | 1.012 (0.891,1.150) | 1.012 (0.914,1.119) | 0.994 (0.885,1.115) | 0.7985 | 0.8326 |
| rs72782676 | 1.148 (0.273,4.822) | 0.779 (0.237,2.565) | 1.264 (0.401,3.990) | 1.352 (0.443,4.125) | 0.704 (0.346,1.430) | 1.379 (0.668,2.846) | 1.298 (0.574,2.933) | 0.8027 | 0.8711 |
| rs4296977 | 0.976 (0.712,1.338) | 0.999 (0.734,1.360) | 1.063 (0.840,1.347) | 1.012 (0.807,1.269) | 1.100 (0.916,1.321) | 0.962 (0.828,1.119) | 0.915 (0.768,1.090) | 0.804 | 0.8803 |
| rs1814576 | 1.133 (0.733,1.750) | 0.877 (0.545,1.410) | 1.224 (0.883,1.695) | 1.126 (0.820,1.547) | 0.938 (0.708,1.244) | 1.139 (0.924,1.403) | 1.129 (0.889,1.433) | 0.8047 | 0.7072 |
| rs74847330 | 1.099 (0.775,1.558) | 0.893 (0.648,1.230) | 1.058 (0.812,1.379) | 1.249 (0.959,1.627) | 1.063 (0.864,1.307) | 1.053 (0.894,1.239) | 1.110 (0.918,1.342) | 0.811 | 0.7029 |
| rs5758343 | 1.044 (0.800,1.364) | 1.104 (0.852,1.429) | 0.986 (0.800,1.214) | 1.051 (0.866,1.275) | 1.137 (0.971,1.331) | 1.129 (0.996,1.279) | 1.014 (0.876,1.174) | 0.813 | 0.5603 |
| rs11236814 | 1.025 (0.688,1.527) | 1.086 (0.727,1.624) | 1.112 (0.811,1.526) | 1.296 (0.945,1.777) | 0.996 (0.785,1.263) | 0.980 (0.813,1.182) | 1.025 (0.825,1.274) | 0.8155 | 0.8575 |
| rs6011033 | 1.206 (0.919,1.581) | 1.003 (0.778,1.294) | 1.174 (0.955,1.444) | 1.007 (0.835,1.215) | 1.042 (0.891,1.219) | 1.119 (0.987,1.270) | 1.073 (0.930,1.238) | 0.8213 | 0.4894 |
| rs10068717 | 1.117 (0.890,1.402) | 0.972 (0.780,1.212) | 1.040 (0.874,1.237) | 1.037 (0.880,1.221) | 0.976 (0.852,1.116) | 1.072 (0.963,1.194) | 0.982 (0.869,1.110) | 0.8222 | 0.8525 |
| rs9372120 | 1.203 (0.934,1.549) | 1.038 (0.801,1.345) | 1.015 (0.828,1.244) | 1.068 (0.883,1.291) | 1.082 (0.925,1.265) | 0.983 (0.866,1.116) | 1.014 (0.878,1.171) | 0.8258 | 0.8401 |
| rs6869502 | 1.039 (0.768,1.406) | 0.885 (0.646,1.213) | 1.092 (0.868,1.373) | 1.170 (0.947,1.447) | 1.079 (0.901,1.293) | 1.015 (0.878,1.174) | 1.036 (0.878,1.223) | 0.8278 | 0.8051 |
| rs10947428 | 1.020 (0.782,1.331) | 0.990 (0.760,1.290) | 1.103 (0.902,1.349) | 1.040 (0.858,1.261) | 0.929 (0.788,1.095) | 1.063 (0.937,1.205) | 1.011 (0.874,1.169) | 0.8433 | 0.8839 |
| rs9573092 | 0.915 (0.731,1.146) | 1.147 (0.913,1.441) | 0.980 (0.823,1.167) | 0.967 (0.820,1.140) | 1.035 (0.902,1.188) | 0.998 (0.896,1.113) | 0.981 (0.867,1.110) | 0.8453 | 0.9109 |
| rs7137828 | 0.976 (0.787,1.211) | 1.140 (0.921,1.411) | 1.075 (0.910,1.270) | 1.146 (0.979,1.341) | 1.085 (0.952,1.236) | 1.028 (0.928,1.140) | 1.072 (0.953,1.206) | 0.8485 | 0.5413 |
| rs10486391 | 0.974 (0.783,1.211) | 1.087 (0.876,1.350) | 0.922 (0.780,1.090) | 1.061 (0.905,1.245) | 0.982 (0.861,1.119) | 0.974 (0.878,1.081) | 1.013 (0.899,1.141) | 0.8518 | 0.9131 |
| rs4145717 | 1.008 (0.798,1.272) | 1.203 (0.961,1.505) | 1.131 (0.948,1.349) | 1.100 (0.931,1.301) | 1.034 (0.899,1.190) | 1.125 (1.008,1.256) | 1.134 (1.000,1.285) | 0.855 | 0.2062 |
| rs35469349 | 1.101 (0.863,1.403) | 1.059 (0.832,1.348) | 1.090 (0.903,1.314) | 1.105 (0.926,1.318) | 0.951 (0.818,1.106) | 1.053 (0.936,1.183) | 1.042 (0.911,1.191) | 0.8573 | 0.8037 |
| rs10865050 | 1.010 (0.742,1.375) | 1.027 (0.757,1.394) | 1.266 (0.980,1.636) | 1.203 (0.949,1.526) | 1.164 (0.958,1.413) | 1.162 (0.997,1.354) | 1.084 (0.913,1.286) | 0.8634 | 0.2654 |
| rs9259819 | 1.051 (0.839,1.315) | 0.973 (0.780,1.214) | 1.099 (0.924,1.306) | 1.053 (0.894,1.240) | 1.085 (0.948,1.242) | 1.134 (1.019,1.263) | 1.047 (0.926,1.183) | 0.8736 | 0.4527 |
| rs72033857 | 0.900 (0.622,1.303) | 0.754 (0.511,1.112) | 1.036 (0.790,1.359) | 1.005 (0.776,1.301) | 0.988 (0.797,1.226) | 0.947 (0.797,1.126) | 0.996 (0.820,1.209) | 0.8808 | 0.9106 |
| rs2988277 | 1.011 (0.809,1.262) | 0.968 (0.778,1.204) | 1.032 (0.869,1.225) | 1.030 (0.876,1.210) | 0.931 (0.815,1.063) | 1.041 (0.936,1.158) | 0.997 (0.884,1.125) | 0.8812 | 0.9347 |
| rs4801001 | 1.076 (0.860,1.346) | 1.186 (0.952,1.479) | 1.157 (0.974,1.374) | 1.081 (0.919,1.273) | 1.066 (0.932,1.220) | 1.073 (0.965,1.194) | 1.026 (0.908,1.160) | 0.8932 | 0.5079 |
| rs16922576 | 0.991 (0.778,1.263) | 1.024 (0.808,1.299) | 0.999 (0.829,1.203) | 0.910 (0.761,1.089) | 0.934 (0.806,1.083) | 1.021 (0.910,1.145) | 0.950 (0.832,1.086) | 0.8957 | 0.9167 |
| rs76081789 | 0.990 (0.619,1.584) | 0.903 (0.579,1.410) | 0.915 (0.645,1.300) | 0.867 (0.627,1.199) | 0.947 (0.717,1.251) | 0.969 (0.775,1.210) | 1.128 (0.861,1.476) | 0.8996 | 0.9375 |
| rs13088318 | 1.069 (0.850,1.344) | 1.149 (0.915,1.444) | 1.187 (0.992,1.420) | 1.055 (0.893,1.245) | 1.030 (0.898,1.181) | 1.062 (0.952,1.184) | 1.081 (0.954,1.225) | 0.9017 | 0.5464 |
| rs78486128 | 1.059 (0.640,1.753) | 0.710 (0.462,1.091) | 0.919 (0.636,1.329) | 0.883 (0.626,1.244) | 0.987 (0.734,1.327) | 0.904 (0.719,1.135) | 0.941 (0.722,1.224) | 0.9022 | 0.8446 |
| rs76167968 | 0.911 (0.616,1.348) | 0.883 (0.603,1.293) | 1.143 (0.823,1.588) | 1.094 (0.806,1.484) | 0.974 (0.764,1.240) | 0.946 (0.783,1.144) | 0.962 (0.774,1.197) | 0.9091 | 0.9462 |
| rs301806 | 1.003 (0.808,1.247) | 1.066 (0.860,1.322) | 0.967 (0.818,1.142) | 1.033 (0.882,1.210) | 1.017 (0.893,1.160) | 0.953 (0.859,1.056) | 1.025 (0.910,1.154) | 0.9121 | 0.955 |
| rs73205303 | 0.928 (0.687,1.253) | 0.841 (0.619,1.141) | 0.851 (0.671,1.080) | 0.991 (0.801,1.227) | 0.857 (0.713,1.031) | 0.937 (0.813,1.080) | 0.929 (0.789,1.094) | 0.9173 | 0.6136 |
| rs7717955 | 1.151 (0.898,1.476) | 0.993 (0.784,1.259) | 1.055 (0.874,1.273) | 1.019 (0.855,1.215) | 1.088 (0.939,1.262) | 0.999 (0.890,1.120) | 1.052 (0.921,1.201) | 0.921 | 0.8925 |
| rs114305667 | 1.060 (0.749,1.498) | 0.964 (0.693,1.340) | 0.895 (0.696,1.152) | 1.040 (0.810,1.336) | 0.973 (0.795,1.192) | 1.032 (0.877,1.215) | 0.925 (0.772,1.108) | 0.9212 | 0.9539 |
| rs63406760 | 0.912 (0.705,1.178) | 1.026 (0.790,1.332) | 1.029 (0.839,1.263) | 1.126 (0.924,1.371) | 1.024 (0.873,1.201) | 1.005 (0.887,1.139) | 1.042 (0.901,1.204) | 0.9213 | 0.9401 |
| rs56129466 | 1.007 (0.772,1.315) | 1.017 (0.782,1.324) | 1.100 (0.891,1.357) | 0.996 (0.821,1.209) | 1.031 (0.877,1.212) | 1.117 (0.981,1.272) | 1.027 (0.887,1.188) | 0.9248 | 0.8477 |
| rs12551834 | 1.148 (0.747,1.762) | 1.251 (0.809,1.935) | 1.007 (0.735,1.380) | 0.948 (0.708,1.268) | 1.001 (0.783,1.280) | 0.969 (0.799,1.175) | 0.959 (0.770,1.194) | 0.9275 | 0.964 |
| rs10663129 | 1.119 (0.891,1.404) | 1.097 (0.877,1.373) | 1.078 (0.904,1.285) | 1.047 (0.887,1.237) | 1.028 (0.895,1.181) | 1.005 (0.901,1.122) | 0.991 (0.874,1.124) | 0.9314 | 0.9321 |
| rs1064213 | 0.934 (0.753,1.159) | 0.969 (0.783,1.198) | 0.951 (0.805,1.123) | 0.984 (0.841,1.152) | 0.957 (0.840,1.090) | 0.939 (0.847,1.041) | 1.027 (0.913,1.156) | 0.9328 | 0.9113 |
| rs2030030 | 0.946 (0.706,1.268) | 1.018 (0.758,1.368) | 1.187 (0.932,1.512) | 1.049 (0.842,1.307) | 1.044 (0.870,1.251) | 1.043 (0.904,1.204) | 1.040 (0.883,1.226) | 0.9486 | 0.9241 |
| rs7130753 | 0.949 (0.749,1.203) | 1.055 (0.831,1.340) | 1.035 (0.860,1.247) | 1.003 (0.843,1.195) | 1.054 (0.911,1.220) | 1.077 (0.960,1.210) | 1.003 (0.880,1.143) | 0.9499 | 0.9332 |
| rs2910162 | 1.051 (0.833,1.326) | 0.997 (0.795,1.252) | 1.046 (0.875,1.251) | 1.086 (0.916,1.286) | 1.040 (0.904,1.196) | 0.978 (0.876,1.091) | 1.030 (0.908,1.169) | 0.9511 | 0.9627 |
| rs11169225 | 1.098 (0.841,1.434) | 0.985 (0.751,1.292) | 1.027 (0.833,1.266) | 1.071 (0.881,1.302) | 1.111 (0.946,1.305) | 1.111 (0.978,1.263) | 1.044 (0.901,1.211) | 0.968 | 0.7693 |
| rs6977955 | 1.169 (0.903,1.515) | 1.015 (0.779,1.323) | 1.102 (0.900,1.350) | 1.028 (0.846,1.249) | 1.015 (0.863,1.193) | 1.046 (0.921,1.189) | 1.048 (0.906,1.212) | 0.9734 | 0.9287 |
| rs59593577 | 1.102 (0.764,1.591) | 0.986 (0.696,1.397) | 0.980 (0.747,1.286) | 1.058 (0.813,1.376) | 1.022 (0.824,1.268) | 1.076 (0.905,1.279) | 0.972 (0.802,1.179) | 0.9806 | 0.9875 |
| rs1837253 | 1.056 (0.823,1.356) | 1.026 (0.804,1.310) | 1.065 (0.878,1.290) | 1.023 (0.854,1.224) | 1.005 (0.866,1.166) | 0.973 (0.866,1.093) | 1.030 (0.900,1.179) | 0.9816 | 0.9915 |
| rs80064395 | 1.014 (0.631,1.628) | 0.919 (0.585,1.442) | 1.021 (0.709,1.472) | 1.123 (0.786,1.604) | 1.014 (0.762,1.348) | 0.942 (0.756,1.175) | 0.977 (0.757,1.260) | 0.9867 | 0.9949 |
| rs4747846 | 1.021 (0.822,1.269) | 1.014 (0.819,1.256) | 1.060 (0.897,1.253) | 1.002 (0.856,1.174) | 1.053 (0.924,1.201) | 1.021 (0.921,1.133) | 1.010 (0.897,1.137) | 0.9973 | 0.9944 |
| rs76991385 | 1.049 (0.838,1.314) | 0.969 (0.774,1.212) | 1.011 (0.849,1.203) | 0.993 (0.842,1.171) | 1.011 (0.882,1.159) | 0.993 (0.892,1.107) | 1.004 (0.888,1.136) | 0.9993 | 0.9998 |

* heterogeneity p value from chi-square test excluding the baseline class of ‘no disease’

† heterogeneity p value from chi-square test including the baseline class of ‘no disease’

Bonferroni corrected p value threshold is 0.0004 (0.05/135). For the six SNPs reported as disease-specific (rs61816761, rs921650, rs115288876, rs12470864, rs6594499 and rs61839660) (9), a less stringent p-value threshold of 0.008 (0.05/6) was also used.

### **Table S6** Pairwise tests among disease latent classes from associations between individual nominal SNPs and profile latent classes in ALSPAC cohort (Table S5).

|  |  | **Atopic March** | **Persistent eczema and wheeze** | **Persistent eczema LO rhinitis** | **Persistent wheeze LO rhinitis** | **Transient wheeze** | **Eczema only** | **Rhinitis only** |
| --- | --- | --- | --- | --- | --- | --- | --- | --- |
|  |  | SNP rs61816761 (*FLG*) | | | | | | |
| **Atopic March** | SNP rs921650 (*GSDMB*) |  | 0.23 | 0.42 | 3.00E-03 | 1.88E-03 | 0.04 | 7.92E-04 |
| **Persistent eczema and wheeze** |  | 0.84 |  | 0.57 | 0.11 | 0.13 | 0.70 | 0.09 |
| **Persistent eczema LO rhinitis** |  | 0.014 | 0.024 |  | 0.015 | 0.011 | 0.18 | 5.25E-03 |
| **Persistent wheeze LO rhinitis** |  | 0.97 | 0.79 | 2.60E-03 |  | 0.79 | 0.09 | 0.86 |
| **Transient wheeze** |  | 0.61 | 0.78 | 8.84E-03 | 0.48 |  | 0.09 | 0.91 |
| **Eczema only** |  | 4.94E-03 | 9.50E-03 | 0.99 | 1.79E-04 | 5.61E-04 |  | 0.05 |
| **Rhinitis only** |  | 0.012 | 0.022 | 0.78 | 1.01E-03 | 3.58E-03 | 0.72 |  |
|  |  | SNP rs11652139 | | | | | | |
| **Atopic March** | SNP rs479844 |  | 0.11 | 0.041 | 0.33 | 0.13 | 8.25E-04 | 0.010 |
| **Persistent eczema and wheeze** |  | 0.69 |  | 0.78 | 0.40 | 0.68 | 0.17 | 0.54 |
| **Persistent eczema LO rhinitis** |  | 0.54 | 0.87 |  | 0.18 | 0.39 | 0.20 | 0.72 |
| **Persistent wheeze LO rhinitis** |  | 0.19 | 0.07 | 0.021 |  | 0.54 | 2.75E-03 | 0.05 |
| **Transient wheeze** |  | 0.57 | 0.29 | 0.14 | 0.29 |  | 6.86E-03 | 0.13 |
| **Eczema only** |  | 0.99 | 0.59 | 0.37 | 0.05 | 0.37 |  | 0.23 |
| **Rhinitis only** |  | 0.13 | 0.04 | 0.007 | 0.92 | 0.17 | 0.010 |  |
|  |  | SNP rs6990534 | | | | | | |
| **Atopic March** | SNP rs5743618 |  | 0.031 | 0.10 | 0.06 | 0.007 | 4.09E-03 | 0.17 |
| **Persistent eczema and wheeze** |  | 0.05 |  | 0.42 | 0.53 | 0.97 | 0.98 | 0.18 |
| **Persistent eczema LO rhinitis** |  | 0.06 | 0.80 |  | 0.81 | 0.27 | 0.23 | 0.59 |
| **Persistent wheeze LO rhinitis** |  | 0.16 | 0.39 | 0.49 |  | 0.38 | 0.34 | 0.40 |
| **Transient wheeze** |  | 4.21E-03 | 0.56 | 0.30 | 0.07 |  | 0.99 | 0.05 |
| **Eczema only** |  | 0.012 | 0.92 | 0.63 | 0.18 | 0.43 |  | 0.022 |
| **Rhinitis only** |  | 0.14 | 0.33 | 0.40 | 0.95 | 0.023 | 0.08 |  |

## MAAS Results

### **Table S7.** Associations between genetic scores and profile latent classes (weighted by membership probabilities) before and after excluding SNPs rs61816761 (*FLG*) and rs921650 (*GSDMB*) in MAAS cohort.

|  | **Multinomial Odds Ratio (95% CI)** | |
| --- | --- | --- |
| **Allergic diseases latent classes** | **Stand. Score** | **Stand. Score excluding *FLG* and *GSDMB*** |
| Atopic March | 1.889 (1.401,2.546) | 1.804 (1.344,2.422) |
| Persistent eczema and wheeze | 1.384 (1.007,1.902) | 1.311 (0.957,1.797) |
| Persistent eczema later-onset rhinitis | 1.354 (1.042,1.759) | 1.349 (1.041,1.749) |
| Persistent wheeze later-onset rhinitis | 1.576 (1.224 2.030) | 1.546 (1.203,1.985) |
| Transient wheeze | 1.115 (0.848,1.465) | 1.104 (0.841,1.449) |
| Eczema only | 1.037 (0.847,1.271) | 1.008 (0.823,1.234) |
| Rhinitis only | 1.316 (1.056,1.604) | 1.290 (1.037,1.604) |
| **P-value for differential association*** | 0.006 | 0.007 |
| **P-value for any association†** | 8.06E-05 | 0.0002 |

Bonferroni corrected significance threshold is 0.000370 (0.05/135)

* Heterogeneity p value from chi-square test excluding the baseline class of ‘no disease’

† Heterogeneity p value from chi-square test including the baseline class of ‘no disease’ (Multinomial Odds Ratio =1)

### **Table S8.** Associations between the top six nominal SNPs (heterogeneity p-value excluding the baseline ‘no disease’ class <0.05 in ALSPAC) and allergic diseases latent classes (weighted by membership probabilities) in MAAS cohort.

|  | **Multinomial Odds Ratio (95% CI)** | | | | | |
| --- | --- | --- | --- | --- | --- | --- |
| SNP[RA] | rs921650[A] | rs61816761[A] | rs11652139[A] | rs479844[G] | rs6990534[A] | rs5743618[C] |
| Nearby Gene | *GSDMB* | *FLG* |  |  |  |  |
| **MAAS** |  |  |  |  |  |  |
| **Allergic diseases latent classes** |  |  |  |  |  |  |
| Atopic March | 1.491 (0.999, 2.225) | 2.107 (0.607, 7.312) | 1.560 (0.999,2.437) | 1.064 (0.713,1.589) | 1.254 (0.812,1.936) | 0.948 (0.604,1.490) |
| Persistent eczema and wheeze | 1.753 (1.132, 2.713) | 1.632 (0.384, 6.924) | 1.806 (1.104,2.955) | 1.110 (0.719,1.712) | 0.805 (0.486,1.334) | 0.808 (0.505,1.291) |
| Persistent eczema later-onset rhinitis | 0.969 (0.679, 1.382) | 1.644 (0.495, 5.469) | 0.934 (0.640,1.363) | 1.108 (0.776,1.581) | 1.121 (0.758,1.657) | 0.984 (0.657,1.475) |
| Persistent wheeze later-onset rhinitis | 1.467 (1.043, 2.062) | 0.670 (0.142, 3.166) | 1.015 (0.705,1.461) | 0.944 (0.672,1.325) | 1.015 (0.694,1.486) | 0.972 (0.660,1.431) |
| Transient wheeze | 1.193 (0.824, 1.727) | 0.830 (0.171, 4.015) | 0.874 (0.589,1.298) | 0.783 (0.540,1.135) | 0.823 (0.535,1.268) | 1.223 (0.780,1.917) |
| Eczema only | 1.322 (1.004, 1.742) | 1.285 (0.462, 3.578) | 1.101 (0.818,1.481) | 1.196 (0.905,1.580) | 1.071 (0.788,1.456) | 0.845 (0.622,1.147) |
| Rhinitis only | 1.171 (0.871, 1.576) | 1.635 (0.585, 4.572) | 1.009 (0.734,1.387) | 0.996 (0.740,1.341) | 0.896 (0.638,1.258) | 1.206 (0.844,1.723) |
| **P-value for differential association*** | 0.298 | 0.883 | 0.122 | 0.567 | 0.655 | 0.554 |
| **P-value for any association†** | 0.054 | 0.861 | 0.153 | 0.668 | 0.761 | 0.664 |

RA=risk allele,; Bonferroni corrected significance threshold is 0.00037 (0.05/135)

* Heterogeneity p value from chi-square test excluding the baseline class of ‘no disease’

† Heterogeneity p value from chi-square test including the baseline class of ‘no disease’ (Multinomial Odds Ratio =1)

# Figures

**
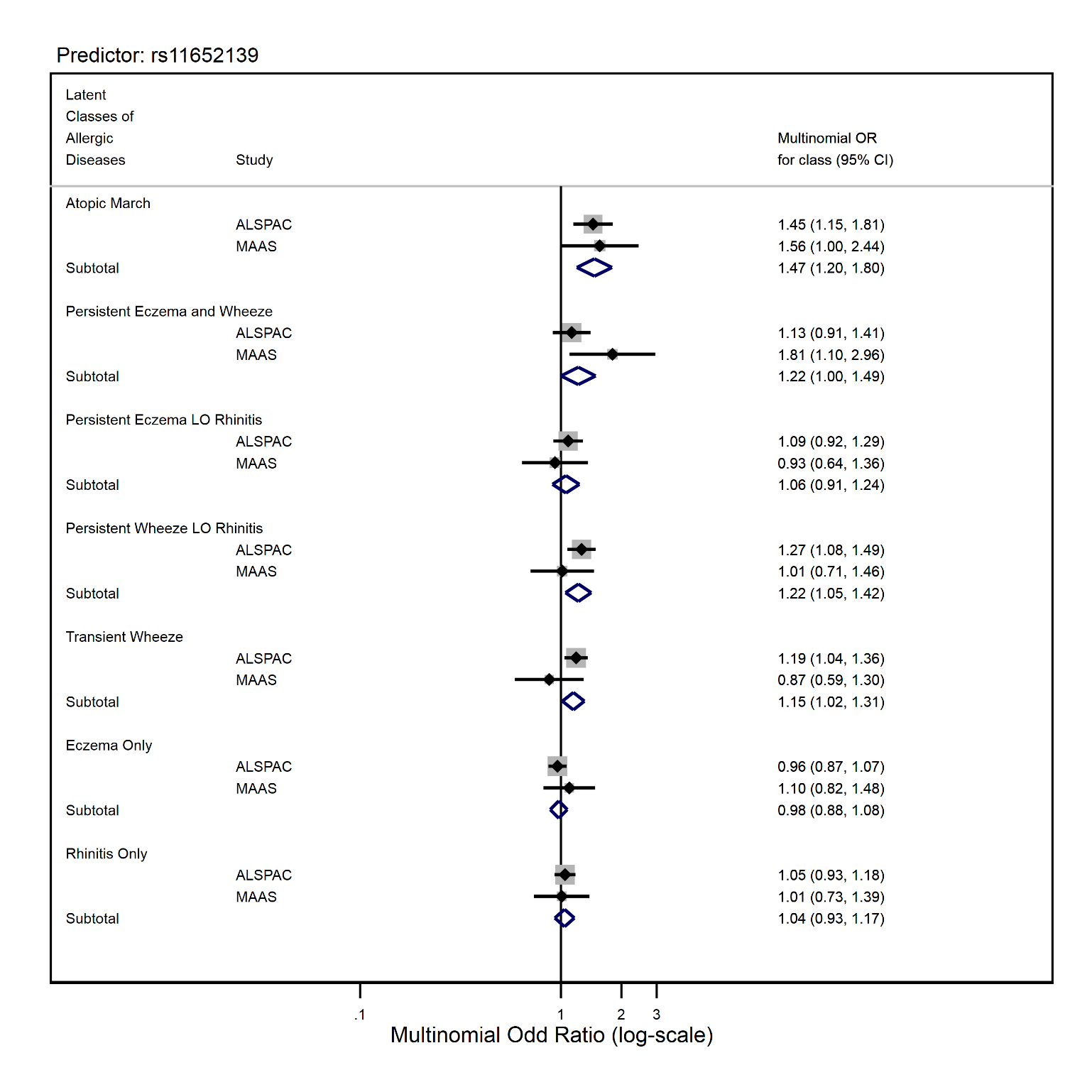
**

### **Figure S1**. Forest plot of the associations between top nominal SNP rs11652139 [A] and allergic disease latent classes in ALSPAC and MAAS cohorts.

###
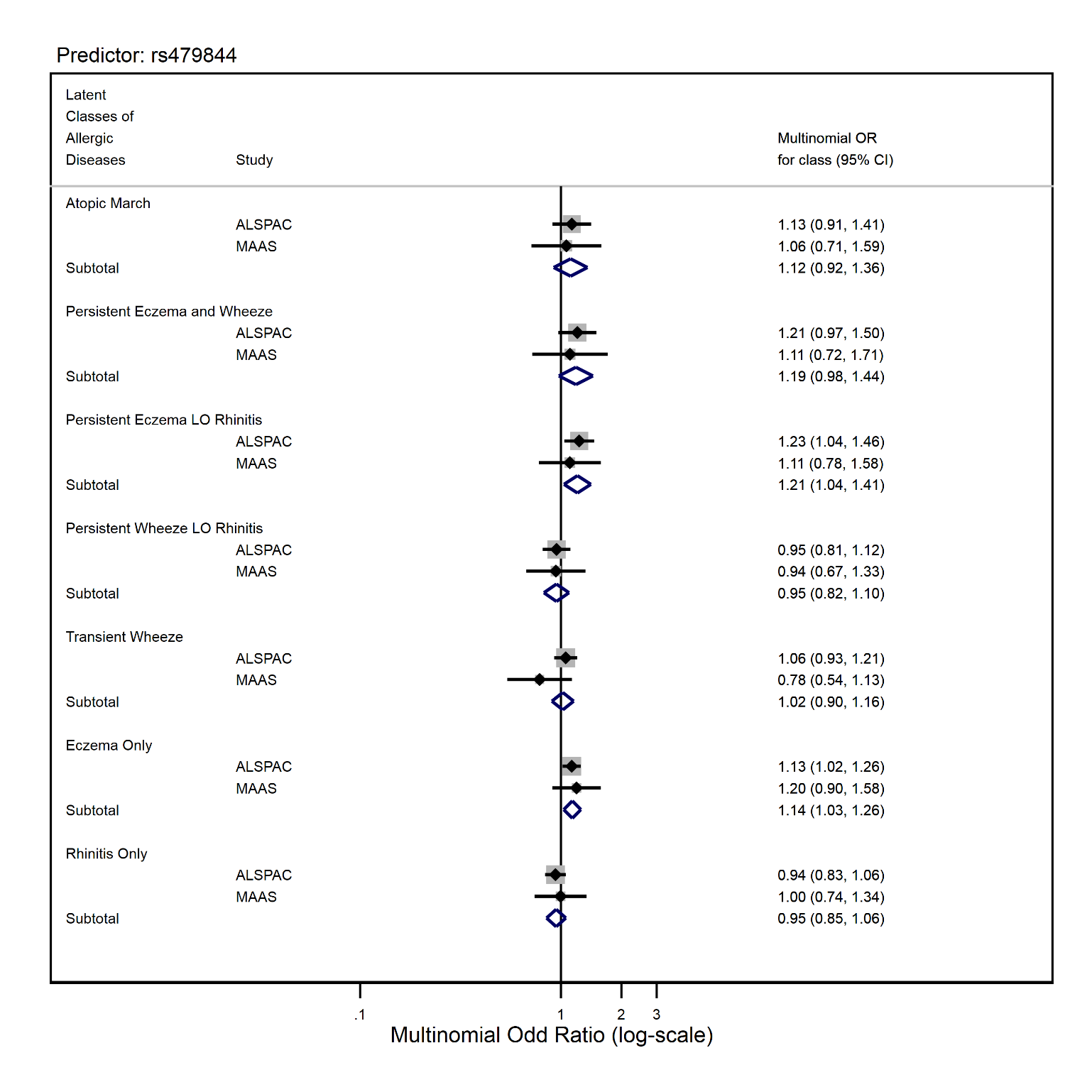


### **Figure S2**. Forest plot of the associations between top nominal SNP rs479844 [G] and allergic disease latent classes in ALSPAC and MAAS cohorts.

###
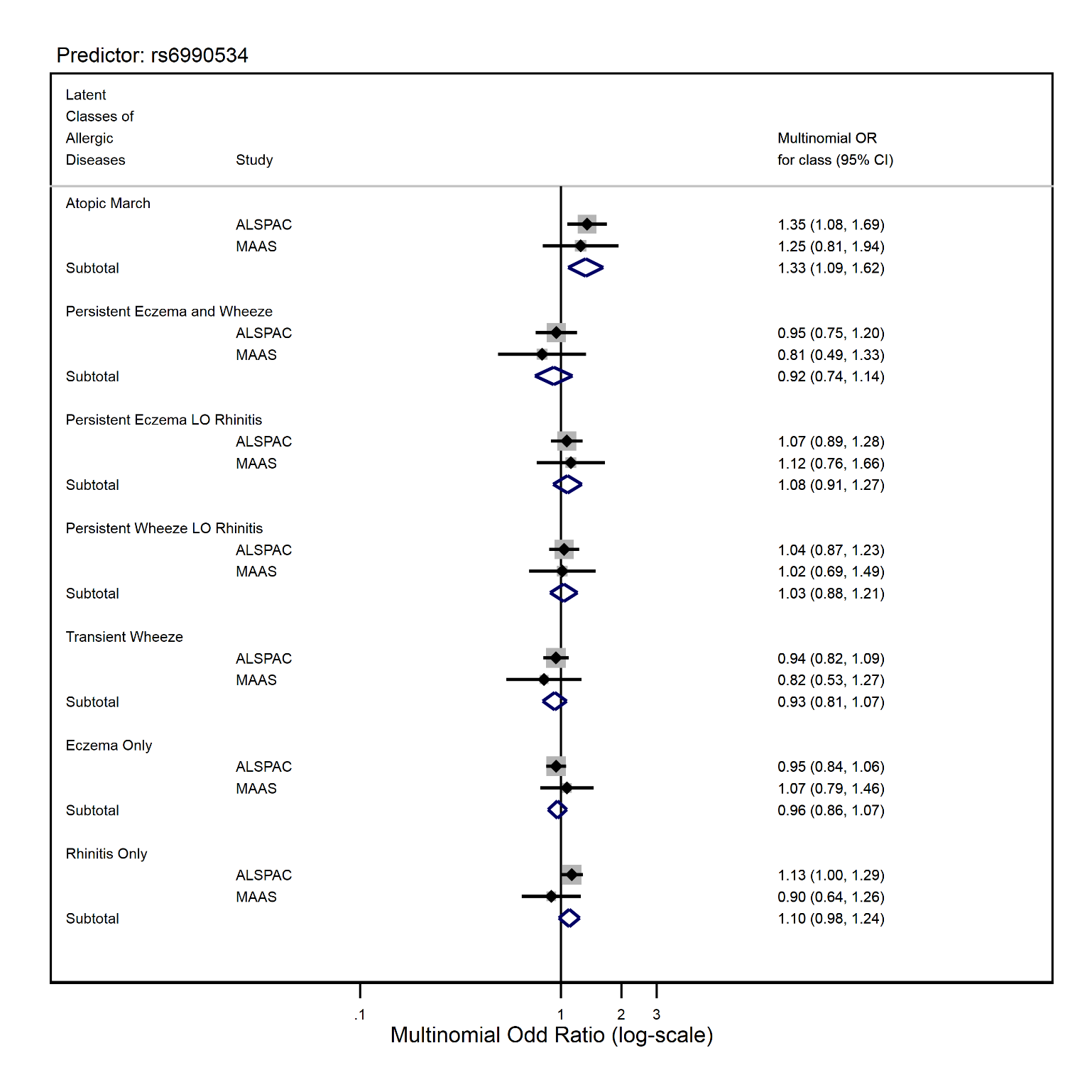


### **Figure S3**. Forest plot of the associations between top nominal SNP rs6990534 [A] and allergic disease latent classes in ALSPAC and MAAS cohorts.

### **
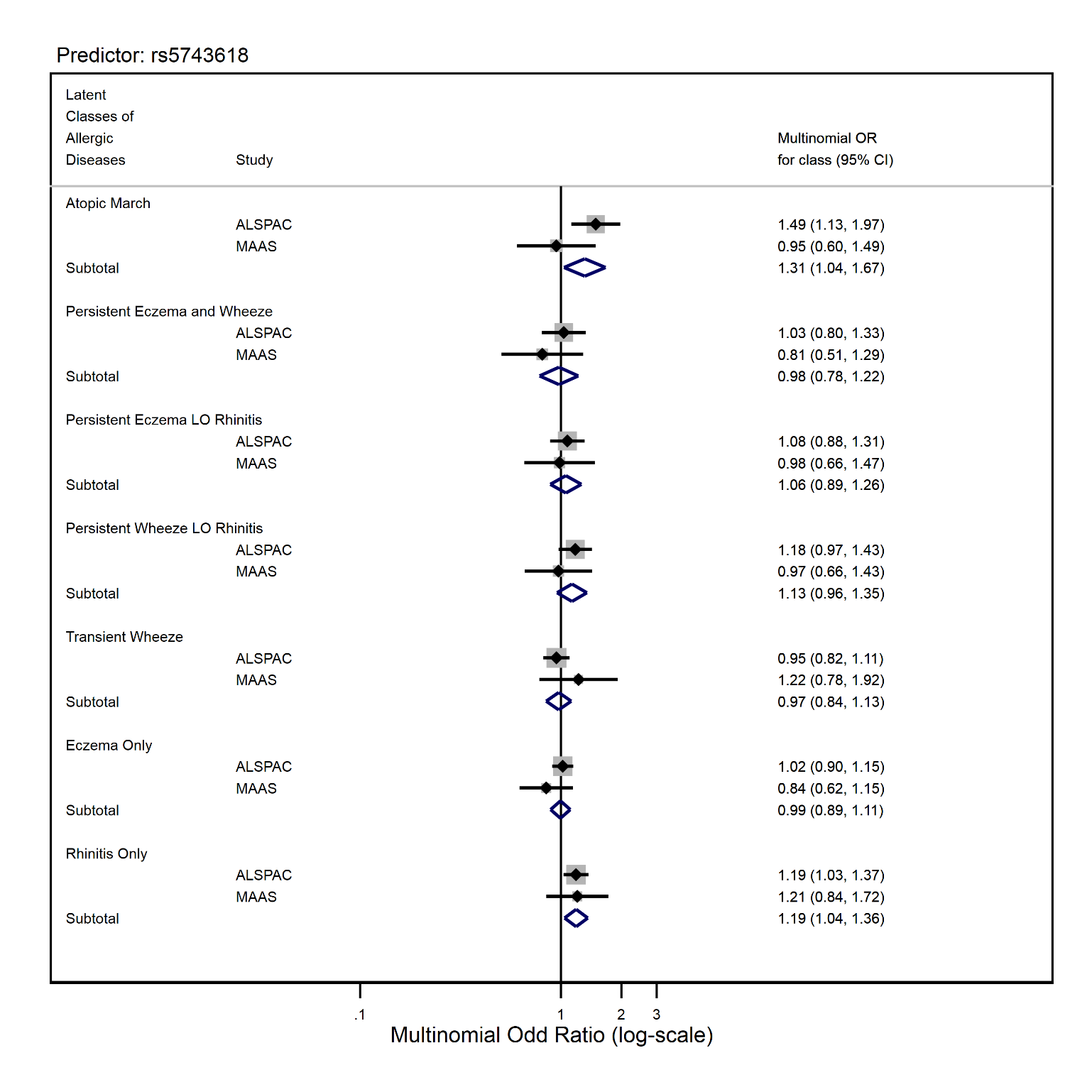
**

### **Figure S4**. Forest plot of the associations between top nominal SNP rs5743618 [C] and allergic disease latent classes in ALSPAC and MAAS cohorts.
